# Furong Tongmai Capsule Ameliorates Atherosclerosis in ApoE ^−/−^ Mice by Modulating Gut Microbiota, Arachidonic Acid Metabolism and Macrophage Polarization

**DOI:** 10.1111/jcmm.71354

**Published:** 2026-09-07

**Authors:** Shuquan Lv, Hanzhou Li, Yuming Wang, Xiuxiu Sang, Feng Wang, Jing Yang, Lirong Fan, Ziang Ma, Lixin Wang, Yuhong Bian, Huantian Cui

**Affiliations:** ^1^ Hebei University of Chinese Medicine Shijiazhuang Hebei China; ^2^ Cangzhou Hospital of Integrated Traditional Chinese Medicine and Western Medicine of Hebei Cangzhou Hebei China; ^3^ Tianjin University of Traditional Chinese Medicine Tianjin China; ^4^ Botou Traditional Chinese Medicine Hospital Botou Hebei China; ^5^ Yunnan University of Chinese Medicine Kunming Yunnan China

**Keywords:** 16S rRNA high‐throughput sequencing, atherosclerosis, Furong Tongmai capsule, macrophage polarization, non‐targeted metabolomics

## Abstract

Furong Tongmai capsule (FRTM) is a traditional Chinese medicine formula with reported lipid‐lowering and anti‐inflammatory activities, but its mechanisms in atherosclerosis (AS) remain unclear. In ApoE^−/−^ mice fed a high‐fat diet, FRTM administration improved serum lipid profiles by reducing total cholesterol, triglycerides and low‐density lipoprotein cholesterol and increasing high‐density lipoprotein cholesterol. FRTM also attenuated aortic lesion formation, reduced pro‐inflammatory cytokines (IL‐6, IL‐1β and TNF‐α), and improved oxidative stress indices. 16S rRNA sequencing showed that FRTM reshaped the gut microbiota, increasing beneficial taxa such as Lactobacillus and Bifidobacterium while decreasing Turicibacter. Functional prediction and untargeted serum metabolomics both suggested that arachidonic acid metabolism was a key pathway affected by FRTM. Furthermore, FRTM was associated with increased p‐PPARγ and EP4 expression, decreased p‐P65, and increased p‐STAT3/STAT3, accompanied by downregulation of M1 markers (iNOS/Nos2) and upregulation of M2 markers (CD206 and ARG1). Faecal microbiota transplantation from FRTM‐treated donors partially recapitulated the anti‐atherosclerotic and anti‐inflammatory effects. Overall, these findings suggest that FRTM may ameliorate AS in a murine model by modulating gut microbiota, arachidonic acid metabolism and macrophage polarization; however, further functional studies are needed to establish causality and assess translational relevance.

## 1 Introduction

Atherosclerosis (AS) represents a chronic, progressive, inflammatory condition affecting the arteries, characterized by lipid deposits and infiltration of white blood cells [1]. AS stands as a significant risk factor for various cardiovascular diseases, including ischemic heart disease, myocardial infarction and stroke [2]. Despite advances in conventional therapies, including lipid‐lowering medications, anti‐inflammatory agents and surgical interventions, the global burden of cardiovascular diseases attributable to AS continues to rise [3]. Furthermore, long‐term use of pharmacological agents may lead to adverse effects such as hepatorenal toxicity and drug tolerance, while surgical interventions remain costly and invasive [4]. Thus, identifying novel therapeutic strategies to address AS remains a critical challenge in modern medicine.

Emerging evidence underscores the critical role of gut microbiota in modulating cardiovascular disease risk, including AS [1, 5]. Metabolomics, a systems biology approach, has revealed that alterations in gut microbiota composition and function are closely associated with metabolic disorders, inflammation and atherosclerotic plaque formation [6]. Traditional Chinese medicine (TCM), with its holistic approach to health, has garnered increasing attention for its potential to mitigate AS through gut microbiota modulation and metabolic pathway regulation. For instance, Ganoderma lucidum polysaccharides have been shown to restore gut microbiota balance and improve lipid metabolism in AS mouse models [7]. Similarly, Dingxin Recipe IV and Guanxin Ning tablets exhibit anti‐AS effects by modulating gut microbiota and their metabolites [8]. These findings highlight the therapeutic potential of TCM in AS treatment and emphasize the need for mechanistic insights into its effects.

Gut dysbiosis can alter host Arachidonic acid (AA) metabolism by directly producing or modifying eicosanoids, via microbial metabolites that modulate host AA‐metabolizing enzymes and through systemic inflammation that releases AA from membrane phospholipids [9]. AA metabolism may represent a crucial interface between gut microbiota dysbiosis and vascular inflammation [10]. AA is released from membrane phospholipids and metabolized through cyclooxygenase (COX), lipoxygenase (LOX) and cytochrome P450 pathways to generate bioactive eicosanoids, including prostaglandins, thromboxanes and leukotrienes. These lipid mediators not only regulate vascular tone and platelet function but also shape innate immune responses. In particular, AA‐derived metabolites such as prostaglandin E2 (PGE2) and 15‐deoxy‐Δ12,14‐prostaglandin J2 (15d‐PGJ2) have been implicated in macrophage polarization through the EP4/STAT3 and PPARγ/NF‐κB signalling axes, thereby influencing inflammatory resolution and atherosclerotic plaque progression [11].

**FIGURE 1:**
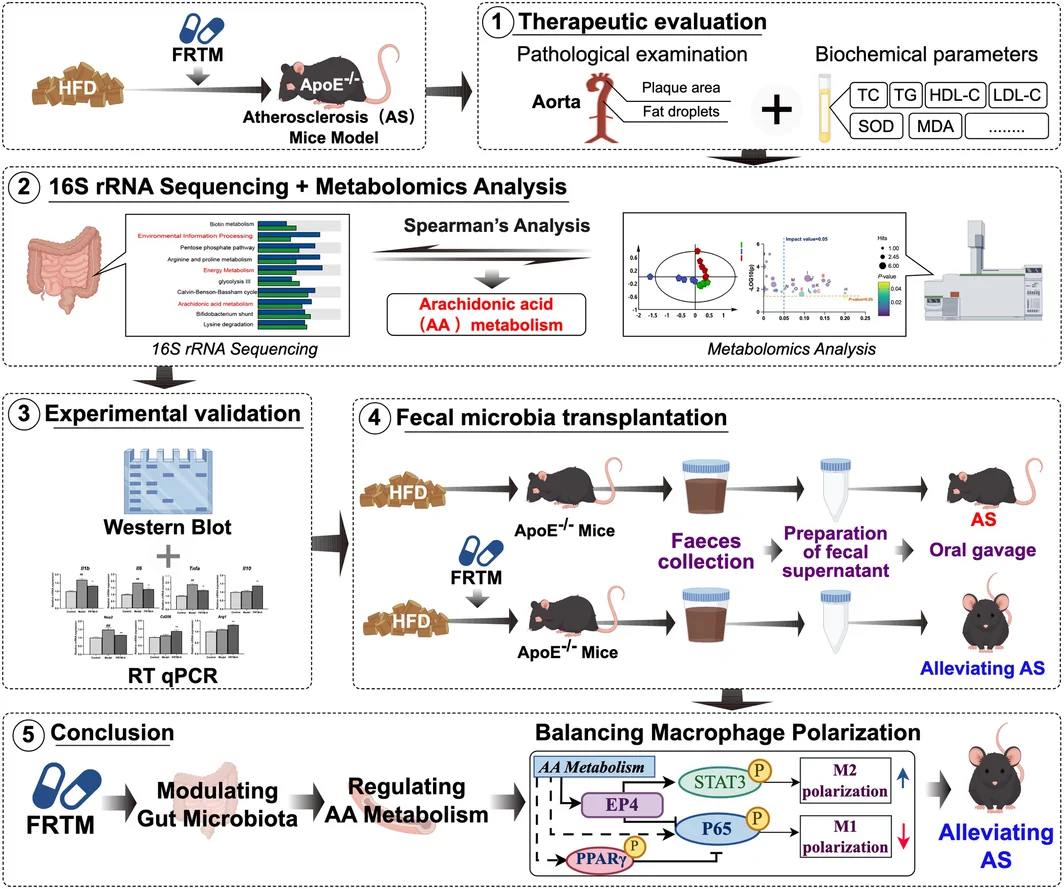
Overall experimental design of this study.

Furong Tongmai capsule (FRTM) comprises *Hirudo*, *Pheretima aspergillum* (E. Perrier), *Mesobuthus martensii* (Karsch), *Astragalus membranaceus* (Fisch.), *Angelica sinensis* (Oliv.) Diels, *Scrophularia ningpoensis* (Hemsl)., *Pueraria montana var. lobata* (Willd.), *Dioscorea nipponica* (Makino), *Polygonum Multiflorum* (Thunb), *Cyathula officinalis* (K. C. Kuan) and *Glycyrrhiza uralensis* (Fisch). In clinical practice, FRTM has shown potential benefits in cardiovascular disease, but its mechanism in AS remains poorly understood [8]. Based on the emerging links among gut microbiota dysbiosis, AA metabolism and macrophage polarization, we hypothesized that FRTM may ameliorate AS by reshaping gut microbiota, thereby modulating AA metabolism and downstream inflammatory signalling. To test this hypothesis, we evaluated the therapeutic effects of FRTM in ApoE^−/−^ mice and integrated histological, biochemical, 16S rRNA sequencing, untargeted metabolomic, signalling and faecal microbiota transplantation analyses to explore the potential microbiota–AA metabolism–macrophage axis underlying the effects of FRTM (Figure 1).

## 2 Materials and Methods

### 2.1 Preparation and Quality Control of FRTM

FRTM (Lot: Z20070041) was purchased from Cangzhou Hospital of Intergrted TCM‐WM Hebei. Ultra‐performance liquid chromatography coupled with mass spectrometer (UPLC–MS) analysis was conducted to detect the major components in FRTM. According to the data obtained from UPLC–MS, the main components identified in FRTM are Astragaloside A from *Astragalus membranaceus* (Fisch.), Ferulic Acid from *Angelica sinensis* (Oliv.) Diels, Harpagide from*Scrophularia ningpoensis* (Hemsl.), Harpagoside from *Mesobuthus martensii* (Karsch.), Puerarin from *Pueraria montana var. lobata* (Willd.), Dioscin from *Polygonum Multiflorum* (Thunb.), Liquiritin from *Glycyrrhiza uralensis* (Fisch.) and Glycyrrhizic acid also from *Glycyrrhiza uralensis* (Fisch.) (Figure S1).

### 2.2 Model Establishment, Grouping and Drug Administration

We procured 10 healthy male SPF‐grade C57BL/6J mice and 50 healthy male SPF‐grade ApoE^−/−^ mice (C57BL/6J strain) from Beijing Huafukang Bio‐Technology Co. Ltd. (licence number SCXK (Beijing) 2020‐0004). These mice were 8 weeks old and weighed between 21 and 23 g upon arrival. They were housed under controlled conditions with a temperature set at 25°C ± 2°C, relative humidity maintained at 50% ± 15% and a 12‐h light/dark cycle. Throughout the study duration, the mice had unrestricted access to water and standard rodent chow. Approval for conducting this experiment was obtained from the Ethics Committee of the Integrated Hospital of Traditional Chinese and Western Medicine in Cangzhou, Hebei Province (CZX2024114). Comprehensive details regarding the reagents and kits employed can be found in the Supporting Information.

After a 1‐week acclimatization period, ApoE^−/−^ mice were fed a high‐fat diet (21% lard, 0.15% cholesterol, 78.85% basal feed) to induce AS. After 8 weeks, the mice were randomly assigned to the model group, atorvastatin group, or three FRTM dose groups using a computer‐generated random sequence, with 10 mice per group. The control and model groups were orally administered saline (0.1 mL/10 g), the atorvastatin group received atorvastatin (10 mg/kg), and the FRTM groups were given FRTM at doses of 5.4, 10.8 and 21.6 g crude herb/kg/day, once daily for 8 weeks. These doses were derived from the clinical human dose and converted to murine equivalent doses based on body surface area normalization using a conversion factor of 9.1 and scaled to account for the lower oral bioavailability and faster metabolism of herbal compounds in rodents [12]. Sample sizes (*n* = 10 per group) were chosen based on previous similar preclinical studies and the amount of tissue required for biochemical, histological, microbiota and metabolomic analyses; however, a formal prospective power calculation was not performed.

According to the experimental design, serum samples from all animals in each group (*n* = 10) were used for biochemical and inflammatory analyses. Six mice per group were randomly selected for histopathological evaluation, and aortic tissues from the remaining animals were used for Western blot analysis. Quantitative histological analysis was performed using predefined criteria and identical imaging settings to minimise operator‐dependent bias.

### 2.3 Serum Biochemical Indicators and Inflammatory Factor Level Detection

After the treatment period, mice were subjected to a 12‐h fasting period, then anaesthetised, and blood samples were collected via cardiac puncture. The blood samples were centrifuged at 1500 ×*g* for 15 min to isolate serum. The serum samples were subsequently analysed for lipid profiles (Triglycerides, TG; Total Cholesterol, TC; High‐Density Lipoprotein Cholesterol, HDL‐C; Low‐Density Lipoprotein Cholesterol, LDL‐C), oxidative stress indicators (Superoxide Dismutase, SOD; Glutathione Peroxidase, GSH‐Px; Malondialdehyde, MDA) and inflammatory cytokines (Interleukin‐6, IL‐6; Interleukin‐1 Beta, IL‐1β; Tumour Necrosis Factor‐Alpha, TNF‐α) using an automated biochemical analyser and ELISA kits following the manufacturer's instructions.

### 2.4 Pathological Staining

Following 12 weeks of treatment, mice were fasted for 12 h, weighed and euthanized. The entire aorta from the aortic root to the iliac bifurcation was carefully excised and fixed in 4% paraformaldehyde. For histological analysis, aortic root tissues were embedded in paraffin and serially sectioned at a thickness of 5 μm. For each mouse, three consecutive sections at comparable anatomical levels were selected at intervals of 50 μm and subjected to haematoxylin and eosin (HE) staining or Oil Red O staining. Images were captured using a light microscope under identical settings. Quantitative analysis of plaque area was performed using ImageJ software. The Oil Red O–positive area (in pixels) from the three sections per mouse was averaged to obtain a representative lesion area for that animal. Due to technical limitations in consistently delineating the total luminal or vessel area across all sections, absolute plaque burden as a percentage of vessel area could not be reliably determined. Therefore, relative plaque area was calculated by normalizing each animal's mean Oil Red O–positive area to the maximum lesion area observed across all animals in the study: Relative lesion area (%) = (Mean Oil Red O area of the individual mouse/Maximum mean Oil Red O area among all mice) × 100. This relative metric was used for intergroup comparisons and statistical analysis.

### 2.5 16S rRNA Sequencing

Building upon prior research [13], 16S rRNA analysis was undertaken. To begin, total genomic DNA was extracted from the cecal contents of the mice utilizing the CTAB/SDS method. Primers were employed to amplify the V3–V4 regions of the gene and conduct PCR reactions. Subsequently, the PCR products underwent purification, electrophoresis and the construction of a sequencing library for sequencing on the Illumina NovaSeq platform. The analysis of sequencing data encompassed diversity analysis, relative abundance analysis at both the phylum and genus levels and microbial functional prediction analysis. Detailed sequencing methodologies and analysis procedures are elaborated upon in the Supporting Information.

### 2.6 Untargeted Metabolomics Analysis

Drawing upon prior investigations [14], untargeted metabolomics analysis was executed. Briefly, 100 μL of each serum sample was mixed with 400 μL of 80% methanol, vortexed, incubated on ice for 5 min, and centrifuged at 15,000 *g* at 4°C for 20 min. The supernatant was diluted with ultrapure water to a final methanol concentration of 53%, centrifuged again at 15,000 *g* at 4°C for 20 min, and the resulting supernatant was used for LC–MS analysis. A pooled QC sample was prepared by mixing equal aliquots from all study samples and injected at regular intervals throughout the analytical sequence to monitor instrument stability. Features with poor reproducibility in QC samples were excluded. Prior to multivariate analysis, the data were normalized, log‐transformed and Pareto‐scaled. PCA was used for unsupervised pattern recognition, and PLS‐DA together with permutation testing was used to assess group separation and model robustness. Detailed LC‐MS conditions, data acquisition, and processing steps are provided in the Supporting Information.

### 2.7 Real‐Time Quantitative PCR (RT‐qPCR) Analysis

RNA was isolated from aortic tissue using Trizol and then converted to cDNA. Gene expression was evaluated via real‐time PCR utilizing the SuperReal PreMix Plus kit. The expression levels were normalized to the *Actb* housekeeping gene using the $2^{-\Delta \Delta C_{T}}$ method. Detailed primer information can be found in Table S2.

### 2.8 Western Blot Analysis

Aortic proteins underwent separation via SDS‐PAGE and were subsequently transferred to PVDF membranes. Following a 1‐h blocking period with skim milk, membranes were exposed to primary antibodies overnight at 4°C. Subsequent to TBST washes, membranes were treated with secondary antibodies for 1 h at room temperature. After the final washing steps and ECL development, bands were detected using a chemiluminescence detector and quantified using ImageJ software.

### 2.9 Faecal Microbial Transplantation (FMT)

#### 2.9.1 The Collection and Prepare of Faeces

The faecal transplantation experiment was performed based on an established protocol [15]. During the FMT procedure, to prepare the stool for gavage, the faeces samples from each of the Model and FRTM‐H groups were first pooled. Subsequently, 100 mg of the mixed sample was resuspended in 1 mL of sterile normal saline. Following this, the suspension underwent vigorous mixing for 10 s and centrifugation at 800 × *g* for 3 min. Afterwards, the supernatant was carefully harvested and served as the transplant material, which was freshly prepared on the day of transplantation, within 10 min prior to administration.

#### 2.9.2 Grouping and Gastric Infusion Method

This transplant material was then orally administered to mice in both the Model→Model group (The faeces from the model group mice were collected and prepared into a suspension, which was administered to the ApoE^−/−^ model mice via oral gavage) and the Model+FRTMH→Model group (The faeces from the FRTM‐H groups' mice were collected and prepared into a suspension, which was administered to the ApoE^−/−^ model mice via oral gavage) (*n* = 10/group) at a dose of 10 mL/kg, twice weekly, for a duration of 8 weeks. Throughout the FMT period, mice from different groups were housed separately to prevent cross‐contamination of their microbial environments.

Recipient ApoE^−/−^ mice were not pretreated with antibiotics or maintained under germ‐free conditions; therefore, donor microbiota engraftment may have been partial, and the FMT results should be interpreted as supportive evidence rather than definitive proof of causality.

### 2.10 Statistical Methods

Statistical analysis was performed using SPSS Statistics 26.0 software. Data are presented as the mean ± standard deviation ($\bar{x}$ ± s). Comparisons between two groups were performed using unpaired Student's *t*‐test. Comparisons among more than two groups were performed using one‐way analysis of variance (ANOVA) followed by Tukey's post hoc test. For metabolomics analysis, PCA and PLS‐DA were used for multivariate pattern recognition, and model robustness was evaluated by permutation testing. Differential metabolites were screened based on VIP > 1, *p* < 0.05 and FC > 1.2 or < 0.8. Spearman correlation analysis was used to assess associations between differential gut microbiota and metabolites. A *p*‐value < 0.05 was considered statistically significant.

## 3 Results

### 3.1 FRTM Treatment Modifies Associated Pathologic Changes and Improves Biochemical Parameters

**FIGURE 2:**
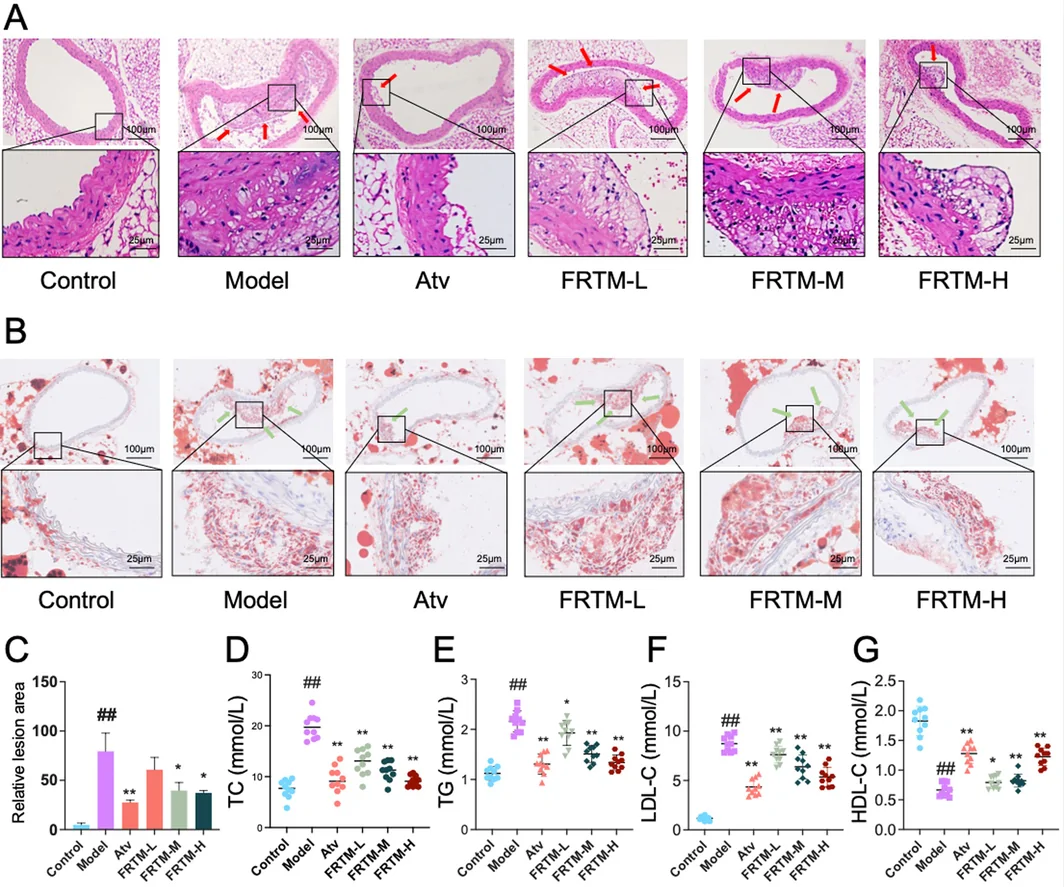
FRTM treatment reduces plaque size and improves lipid levels. (A–C) HE staining showed that FRTM treatment could improve the pathological changes in the aorta (A) and oil‐red O staining indicated that FRTM could decrease the plaque area and fat droplets in the aorta of AS mice (B, C). (D–G) FRTM decreased the levels of TC (D) and TG (E) in serum of AS mice and improved the abnormal LDL‐C (F) and HDL‐C (G) levels in serum of AS mice. Data are presented as the mean ± SD, *n* = 6 for pathological detection. *n* = 10 for biochemical testing. ^##^ *p* < 0.01 and ^#^ *p* < 0.05 as compared to the Control group; **p < 0.01 and *p < 0.05, as compared to the Model group. Representative histological images are shown, and quantitative analyses were performed by averaging multiple sections from six mice per group.

We found through histological examination that compared to the Control group, the aortic tissue of Model group mice displayed atherosclerotic lesions, characterized by endothelial damage, fibrous plaques, calcification and local inflammatory infiltration. In contrast, the Atorvastatin group and all FRTM groups exhibited varying degrees of improvement in the aortic lesion area. These improvements included reduced endothelial cell shedding, a more intact structure, regular smooth muscle cell morphology, diminished formation of fibrous plaques and calcification and absence of obvious inflammatory infiltration (Figure 2A). Oil Red O staining demonstrated that the Model group had an irregular vascular lumen with evident atherosclerotic plaques, inflammatory cell infiltration within the plaques and significant thickening of the vascular intima at the site of plaque attachment, characterized by extensive red staining (Figure 2B). Following intervention with Atorvastatin and FRTM at different doses, although a small amount of atherosclerotic plaques remained visible, there was improvement in plaque area and lipid droplets to varying extents. Quantitative analysis revealed a significantly higher relative lesion area in the Model group compared to controls. Treatment with Atorvastatin and FRTM at different doses reduced both the size and intensity of lipid staining, with corresponding decreases in relative lesion area, demonstrating a dose‐dependent attenuation of atherosclerotic plaque burden (Figure 2C). Furthermore, we assessed the therapeutic effects of FRTM on AS mice by measuring the levels of TC, TG, LDL‐C and HDL‐C in the serum of mice from each group (Figure 2D–G). The Model group exhibited significantly elevated levels of serum TC, TG and LDL‐C compared to the Control group, alongside a notable decrease in HDL‐C levels. Intervention with Atorvastatin and FRTM at low, middle and high doses significantly reduced the serum levels of TC, TG and LDL‐C, while increasing the levels of HDL‐C in Model mice. These findings collectively suggest the therapeutic potential of FRTM in AS mice.

### 3.2 FRTM Inhibits the Levels of Oxidative Stress and Inflammatory Factors in AS Mice

**FIGURE 3:**
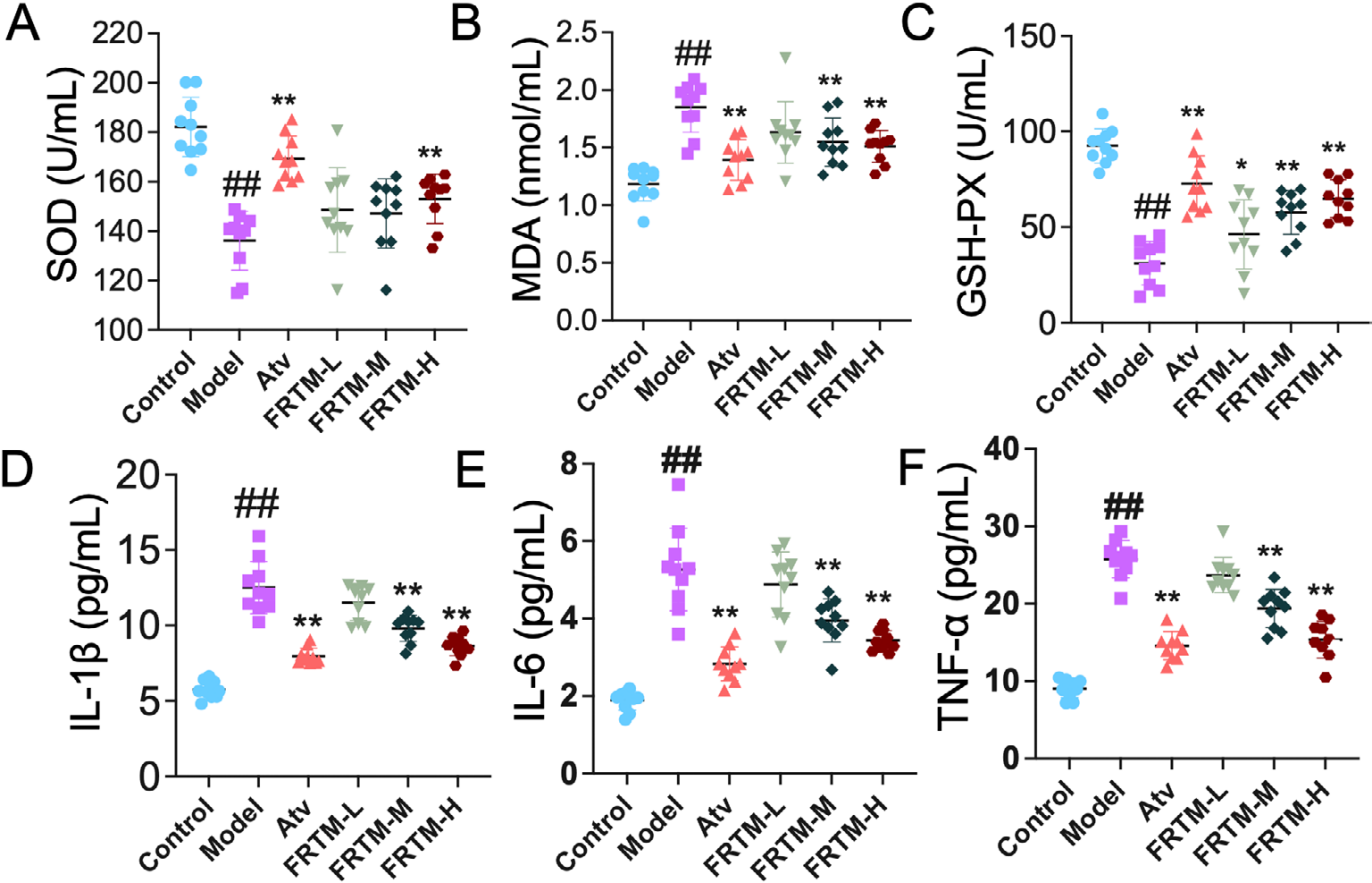
FRTM suppresses the oxidative stress and inflammatory reactions of AS mice. (A‐C) FRTM treatment improved the levels of oxidative stress‐related indicators, including SOD (A), MDA (B) and GSH‐PX (C) in serum. (D–F) FRTM treatment significantly improved inflammatory factors, as seen in the significant reduction of IL‐1 β (D), IL‐6 (E) and TNF‐α (F) levels in serum after FRTM treatment. *n* = 10 per group.

We further examined FRTM's impact on oxidative stress in AS mice by assessing the activities of SOD and GSH‐Px. We also assessed the levels of MDA in the serum of each group's mice (Figure 3A–C). The Model group displayed significantly reduced SOD and GSH‐Px activities, along with elevated MDA levels compared to the Control group. However, intervention with Atorvastatin and FRTM at middle and high doses significantly boosted the activities of SOD and GSH‐Px while reducing MDA levels in the serum of AS mice compared to the Model group.

Furthermore, we examined FRTM's impact on the inflammatory response in the serum of AS mice by measuring the levels of pro‐inflammatory cytokines IL‐6, IL‐1β, and TNF‐α using ELISA (Figure 3D–F). Compared to the Control group, the Model group exhibited significantly elevated levels of IL‐6, IL‐1β and TNF‐α in the serum. However, intervention with Atorvastatin and FRTM at middle and high doses significantly decreased the levels of these pro‐inflammatory factors in the serum of Model group mice. These findings suggest that FRTM may mitigate AS oxidative stress and inflammatory responses by modulating the levels of associated factors.

### 3.3 FRTM Therapy Affected the Gut Microbiota of AS Mice

**FIGURE 4:**
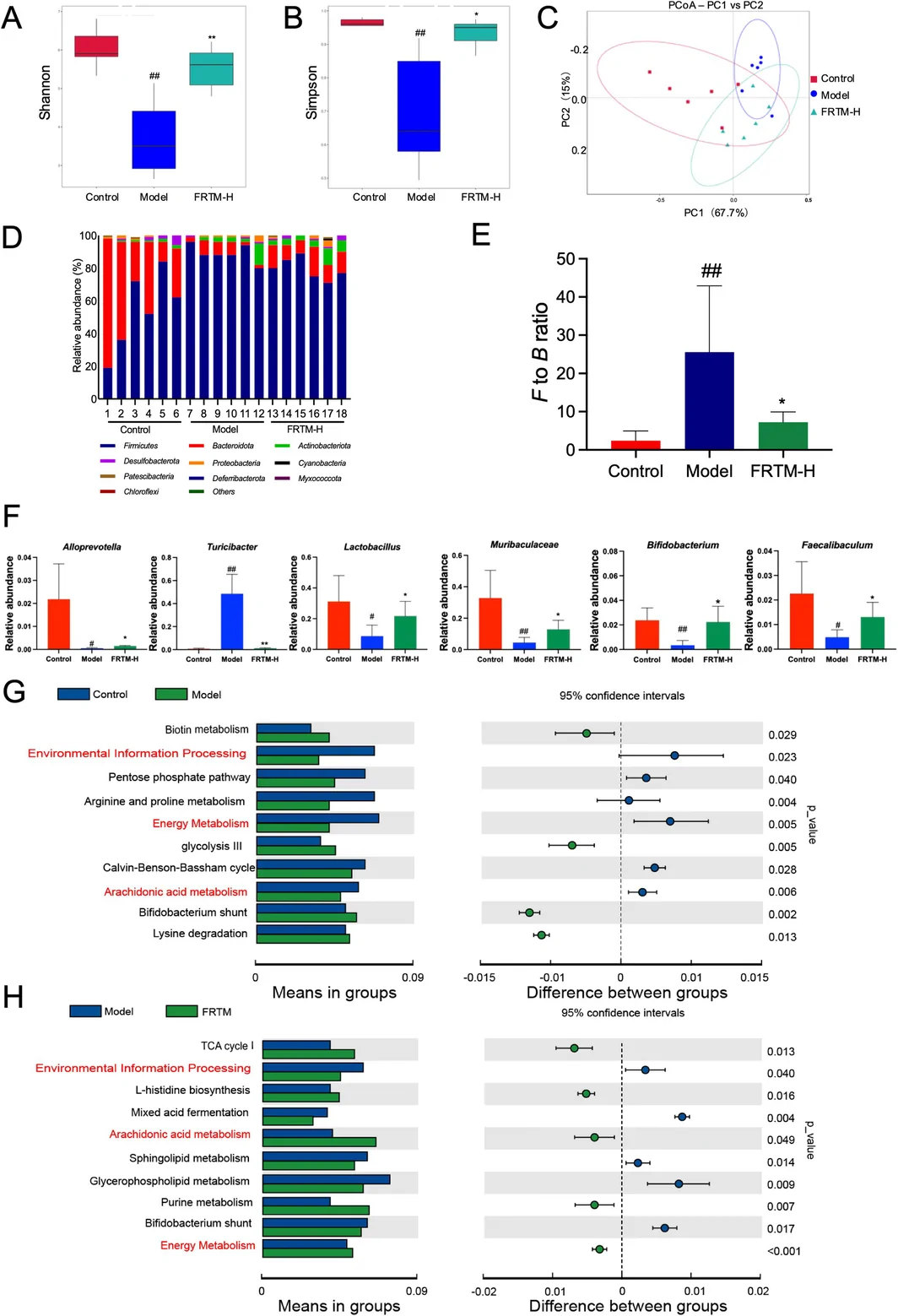
FRTM therapy affected the gut microbiota of AS mice based on 16S rRNA sequencing. (A, B) Shannon and Simpson indexes decreased in AS mice after FRTM treatment. (C) PCoA showed distinct β diversity of gut microbiota in each group. (D, E) The *F* to *B* ratio was decreased in AS model mice after FRTM‐H treatment (at the phylum level). (F) At the genus level, FRTM treatment affected the relative abundances of *Latobacillus*, *Muribaculaceae*, *Alloprevotella*, *Bifidobacterium*, *Faecalibaculum* and *Turicibacter* in AS mice. (G, H) Results of the bacterial function prediction showing that FRTM mainly regulated Environmental Information Processing, Energy Metabolism and AA metabolism. Common pathways between Model vs. Control and FRTM‐H vs. Model are highlighted in red. *n* = 6 per group.

This study employed 16S rRNA high throughput sequencing to explore the impact of FRTM on the gut microbiota in AS mice. Alpha diversity, indicating within‐sample diversity, was significantly affected by AS but restored by FRTM. Beta diversity was evaluated using principal coordinate analysis (PCoA) and clustering based on the Bray‐Curtis distance. PCoA revealed clear distinctions between model group samples and normal samples, while FRTM group samples exhibited closer proximity to normal samples (Figure 4A,B). Cluster analysis corroborated these findings, demonstrating the ability of FRTM to effectively reverse gut microbiota alterations in AS mice (Figure 4C).

The Figure 4D,E illustrates the composition of the gut microbiota at the phylum level in each sample group, with Firmicutes and Bacteroidetes predominating. Compared to the Control group, the Firmicutes/Bacteroidetes (*F* to *B*) ratio was significantly elevated in the Model group (*p* < 0.01), whereas it was significantly reduced in the FRTM group compared to the Model group (*p* < 0.05). At the genus level, the Model group exhibited relatively increased abundance of *Turicibacter* compared to the Control group. Conversely, the abundance of *Lactobacillus, Muribaculaceae, Bifidobacterium, Faecalibaculum*, and *Alloprevotella* was relatively decreased in the Model group compared to the Control group. In comparison with the Model group, the FRTM group showed relatively increased abundance of *Lactobacillus, Muribaculaceae, Bifidobacterium, Faecalibaculum and Alloprevotella*, while the abundance of *Turicibacter* was relatively decreased (Figure 4F).

### 3.4 Microbiota Functional Prediction Analysis

To investigate the functional changes in the gut microbiota of AS mice following FRTM‐H intervention, we utilized PICRUSt2 analysis to predict alterations in microbiota function among the Control and Model groups, as well as between the Model and FRTM‐H groups. The Figure 4G,H outline the top 10 metabolic pathways exhibiting significant differences in proportion.

### 3.5 FRTM Regulates a Variety of Metabolites in AS Mice

**FIGURE 5:**
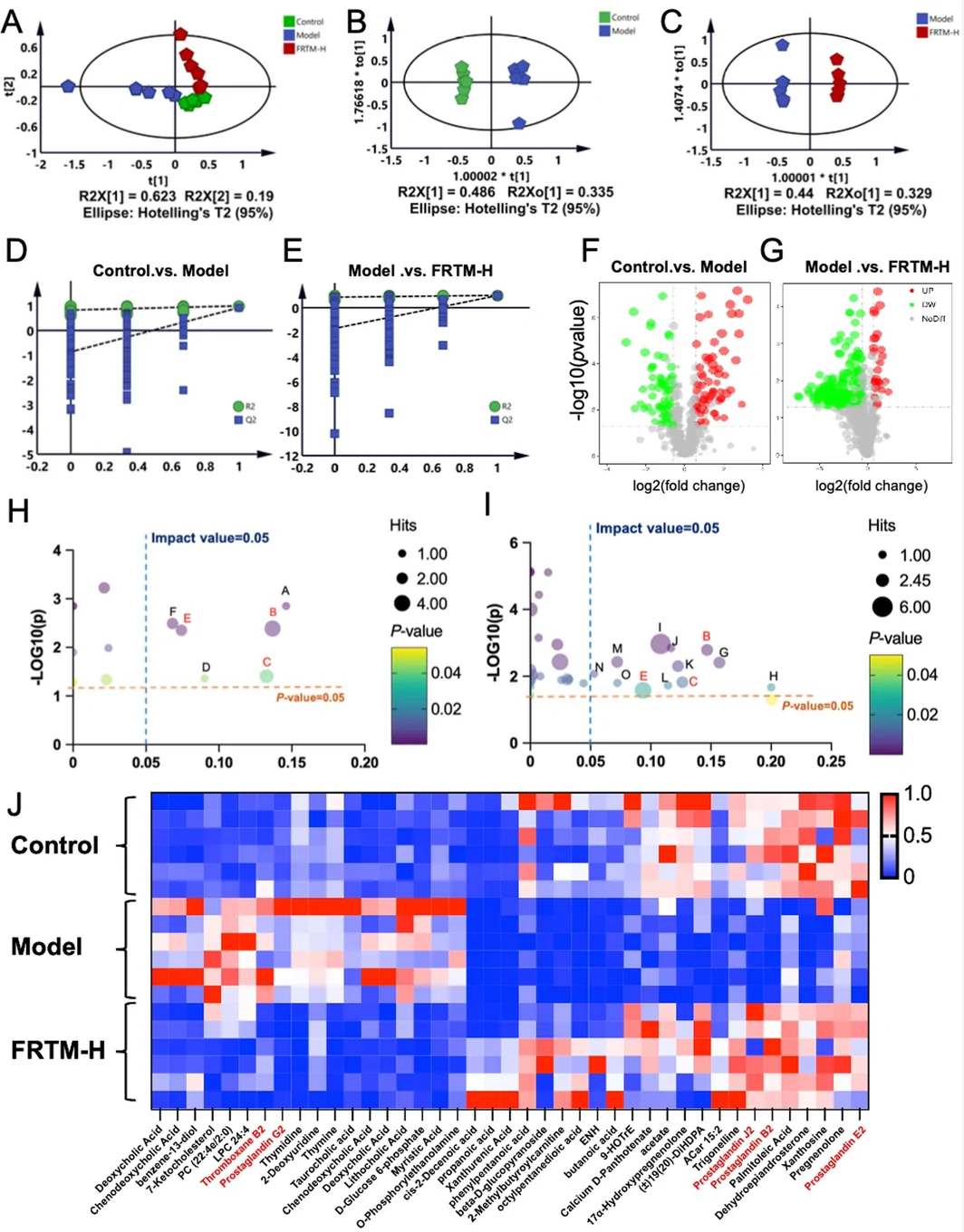
Non‐targeted metabolomics indicated that FRTM treatment regulated the metabolites in serum of AS mice, and correlations with gut microbiota were provided. (A) PCA results showing good differentiation between the Control group, Model group and FRTM‐H group. (B–E) PLS‐DA model used to identify differential metabolites and permutation test used to show that the model was reliable. (F, G) Volcano plot showed the differentially regulated metabolites between Model and Control, as well as between FRTM‐H and Model groups. (H, I) Pathway analysis between Model and Control, as well as between FRTM‐H and Model. (J) The expression of common differential metabolites among Control, Model, and FRTM‐H groups was presented using a heatmap. A, starch and sucrose metabolism; B, AA metabolism；C, pyrimidine metabolism; D, histidine metabolism; E, steroid hormone biosynthesis; F, purine metabolism; G, tryptophan metabolism; H, cysteine and methionine metabolism; I, alanine, aspartate and glutamate metabolism; J, primary bile acid biosynthesis; K, tyrosine metabolism; L, lysine degradation; M, arginine and proline metabolism; N, porphyrin metabolism; O, pentose and glucuronate interconversions. The common pathways between Model vs. Control and FRTM‐H vs. Model are highlighted in red. *n* = 6 per group.

We utilized principal component analysis (PCA) to distinguish metabolomic data between groups by generating new variables from combinations of metabolites. As shown in Figure 5A, notable differences were evident in the coordinate values along both the X and Y axes when comparing the model and Control groups, as well as between the FRTM‐H and model groups. To differentiate metabolites, we employed a PLS‐DA model, and its performance was assessed through a permutation test, providing *R* ^2^ and *Q* ^2^ values to indicate model stability and predictive capability (Figure 5B–E).

We identified differential metabolites using a VIP value cutoff of *p* ≤ 0.05 and VIP where VIP > 1, combined with a fold change (FC) threshold of > 1.2 or < 0.8. Details of differential metabolites were provided in the Supporting Information. Volcano plot showed the differentially regulated metabolites between Model and Control, as well as between FRTM‐H and Model groups (Figure 5F,G). These metabolites underwent analysis in MetaboAnalyst 5.0 for metabolic pathway enrichment. Interestingly, untargeted metabolomics results demonstrated that FRTM treatment regulated AA metabolism, Pyrimidine metabolism and Steroid hormone biosynthesis (Figure 5H,I). Furthermore, we used a heatmap to demonstrate the expression of commonly differential metabolites among Control, Model and FRTM‐H groups (Figure 5J).

**FIGURE 6:**
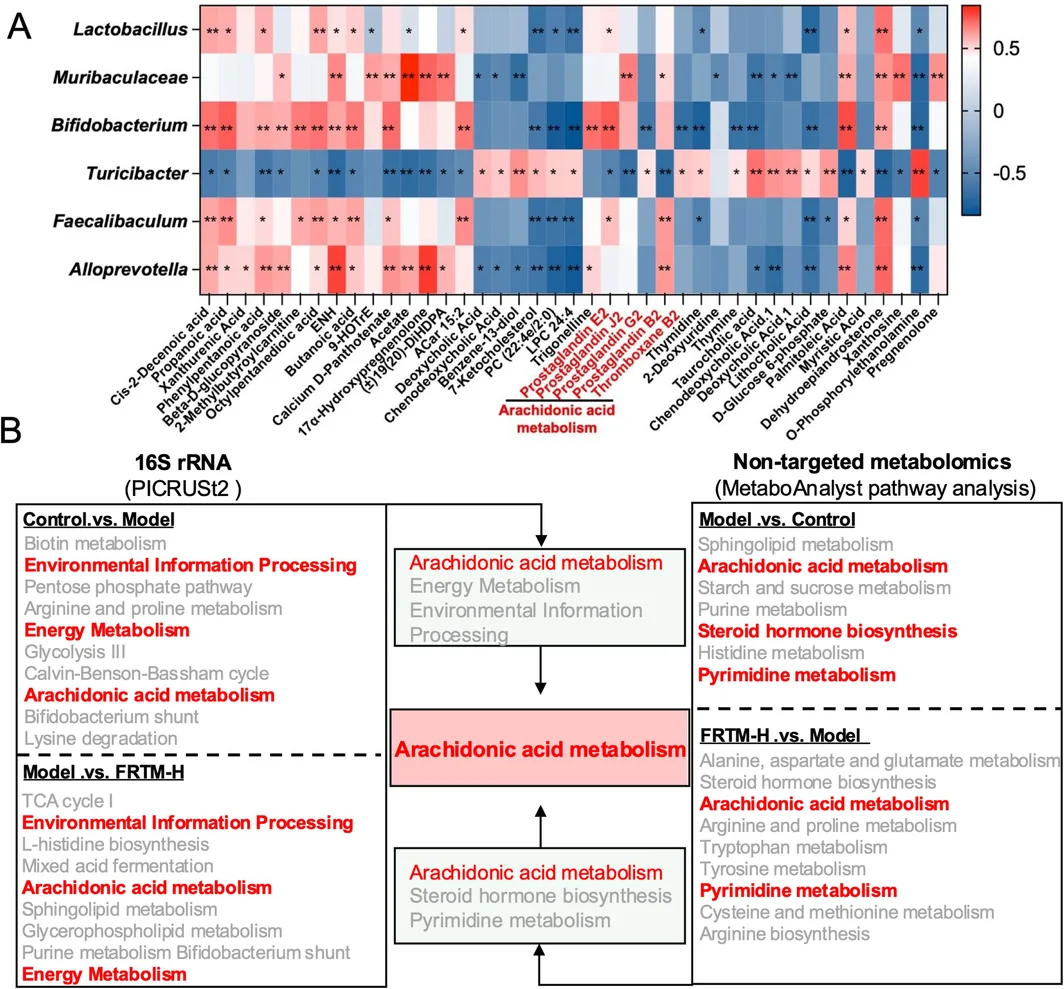
Integrated analysis of gut microbiota and serum metabolites identified arachidonic acid (AA) metabolism as a shared pathway associated with FRTM‐H treatment in AS mice. (A) Spearman correlation heatmap showing significant associations between differential gut microbiota and selected metabolites; only statistically significant correlations are shown. (B) Overlap analysis of PICRUSt2‐based functional prediction and untargeted metabolomic pathway enrichment identified arachidonic acid metabolism as the common pathway among the Control, Model and FRTM‐H groups.

Moreover, Spearman's correlation analysis indicated correlations between *Lactobacillus, Muribaculaceae, Bifidobacterium, Turicibacter, Faecalibaculum, Alloprevotella* and the majority of metabolites (Figure 6A). 16S rRNA and untargeted metabolomics identified that the shared metabolic pathway was AA metabolism, among the Control, Model and FRTM‐H groups (Figure 6B).

### 3.6 FRTM Regulates the PPARγ/NF‐κB and EP4/STAT3 Pathways to Inhibit the Expression of Inflammatory‐Related Factors

**FIGURE 7:**
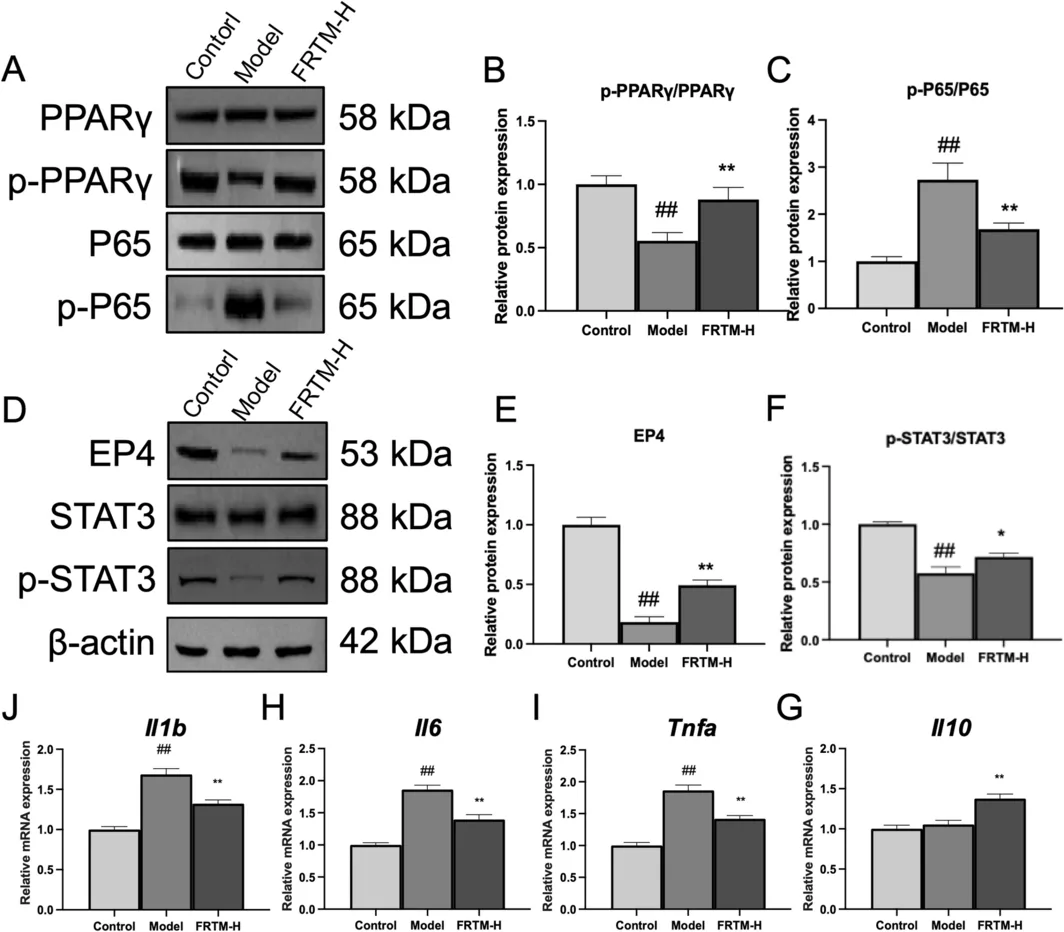
FRTM altered the PPARγ/NF‐κB pathway and EP4/STAT3 pathway to reduce inflammatory reactions. (A–F) Western blot results showed that FRTM intervention increased the expression of p‐PPARγ/PPARγ and reduced the expression of p‐P65/P65; meanwhile, the expressions of EP4 and p‐STAT3/STAT3 were increased after FRTM intervention. (G–J) RT‐qPCR results showed that FRTM treatment reduced *Il1b, Il6* and *Tnfa* mRNA levels and increased *Il10* mRNA level. *n* = 3 per group.

Recent studies indicate that metabolites of arachidonic acid, such as PGE2 and PGJ2, modulate inflammation through the PPARγ/NF‐κB and EP4/STAT3 signalling pathways [16]. These pathways regulate macrophage polarization, which is crucial in the progression of AS [11]. Specifically, PGE2 activates the EP4 receptor to promote STAT3 phosphorylation, while PGJ2 activates PPARγ to inhibit NF‐κB activity, reducing inflammatory cytokine production [17]. Consequently, these metabolites are involved in regulating plaque formation and progression in AS. Our metabolomics data illustrate that FRTM can alter levels of AA metabolites, elevating prostaglandin E2 (PGE2), prostaglandin J2 (PGJ2) and prostaglandin B2 (PGB2) while reducing prostaglandin G2 (PGG2) and (thromboxane B2) TXB2. To delve deeper into this mechanism, we analysed the impact of FRTM on the PPARγ/NF‐κB and EP4/STAT3 pathways using Western blotting. The results indicated that FRTM treatment increased p‐PPARγ levels and decreased p‐P65 phosphorylation compared to the untreated model group. Additionally, compared to the model group, FRTM elevated EP4 levels and increased p‐STAT3/STAT3 levels (Figure 7A–F). Further analysis via RT‐qPCR revealed that the model group exhibited elevated *Il1b, Il6* and *Tnfa* levels alongside reduced *Il10* compared to controls. Treatment with FRTM normalized these cytokine levels, diminishing pro‐inflammatory *Il1b, Il6* and *Tnfa* while augmenting anti‐inflammatory *Il10* (Figure 7G–J). These results suggest that FRTM may alleviate inflammation through modulation of the PPARγ/NF‐κB and EP4/STAT3 pathways.

### 3.7 FRTM Inhibits Macrophage Polarization

**FIGURE 8:**
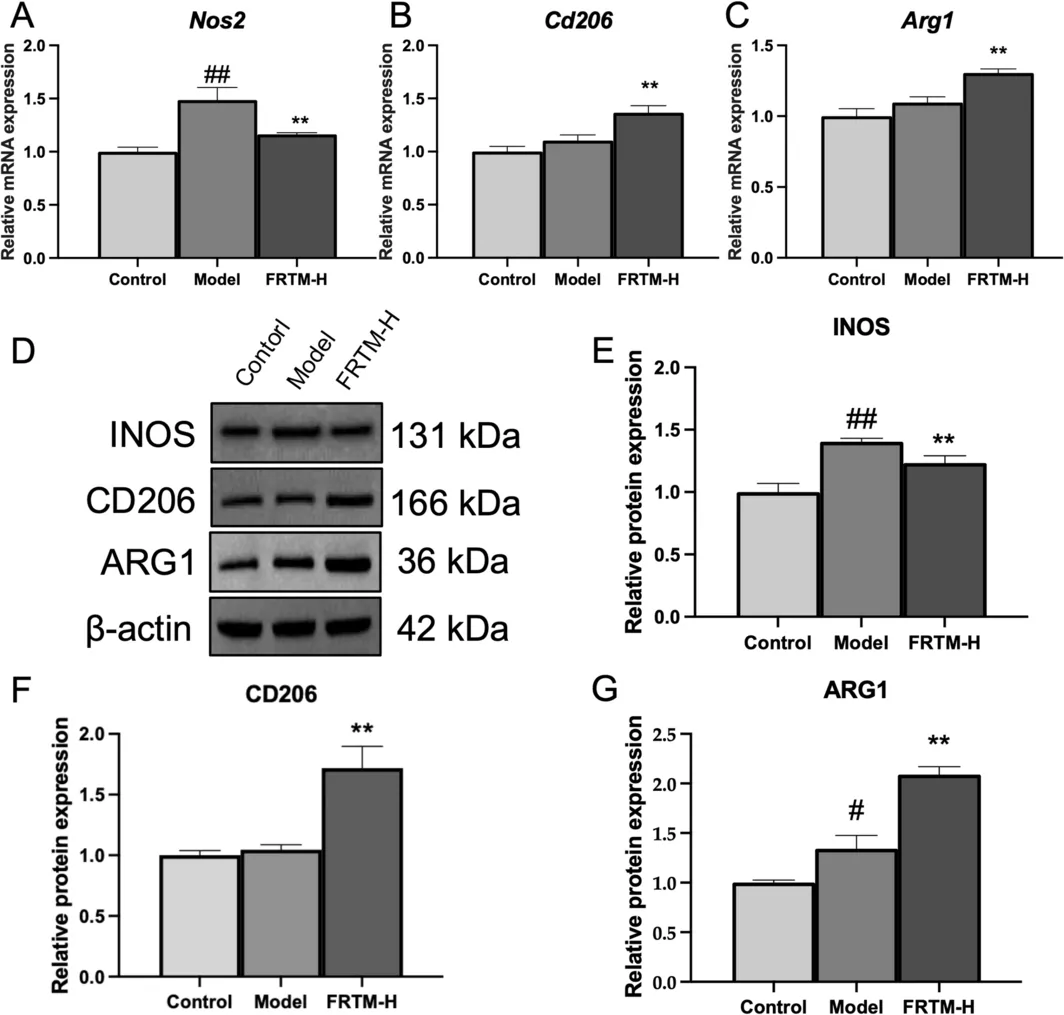
FRTM treatment regulated macrophage polarization. (A–C) RT‐qPCR results showed that FRTM treatment downregulated the expression of *Nos2*, while upregulating the expressions of *Cd206* and *Arg1*. (D–G) Western blot showing that FRTM treatment downregulated the expression of INOS, while upregulating the expressions of CD206 and ARG1. *n* = 3 per group.

Because PPARγ/NF‐κB and EP4/STAT3 signalling have been implicated in macrophage polarization in AS, we examined whether FRTM‐associated signalling changes were accompanied by alterations in macrophage phenotype. RT‐qPCR showed that FRTM treatment downregulated Nos2 while upregulating Cd206 and Arg1 expression (Figure 8A–C). Western blot analysis showed that FRTM treatment decreased iNOS expression while increasing CD206 and ARG1 protein levels compared with the model group (Figure 8D–J). These results are consistent with a shift toward an M2‐like macrophage phenotype, although they do not establish direct causality.

### 3.8 FMT (Model+FRTM‐H → Model Group) Partially Improves AS‐Related Pathology, Biochemical Parameters, Oxidative Stress and Inflammation

**FIGURE 9:**
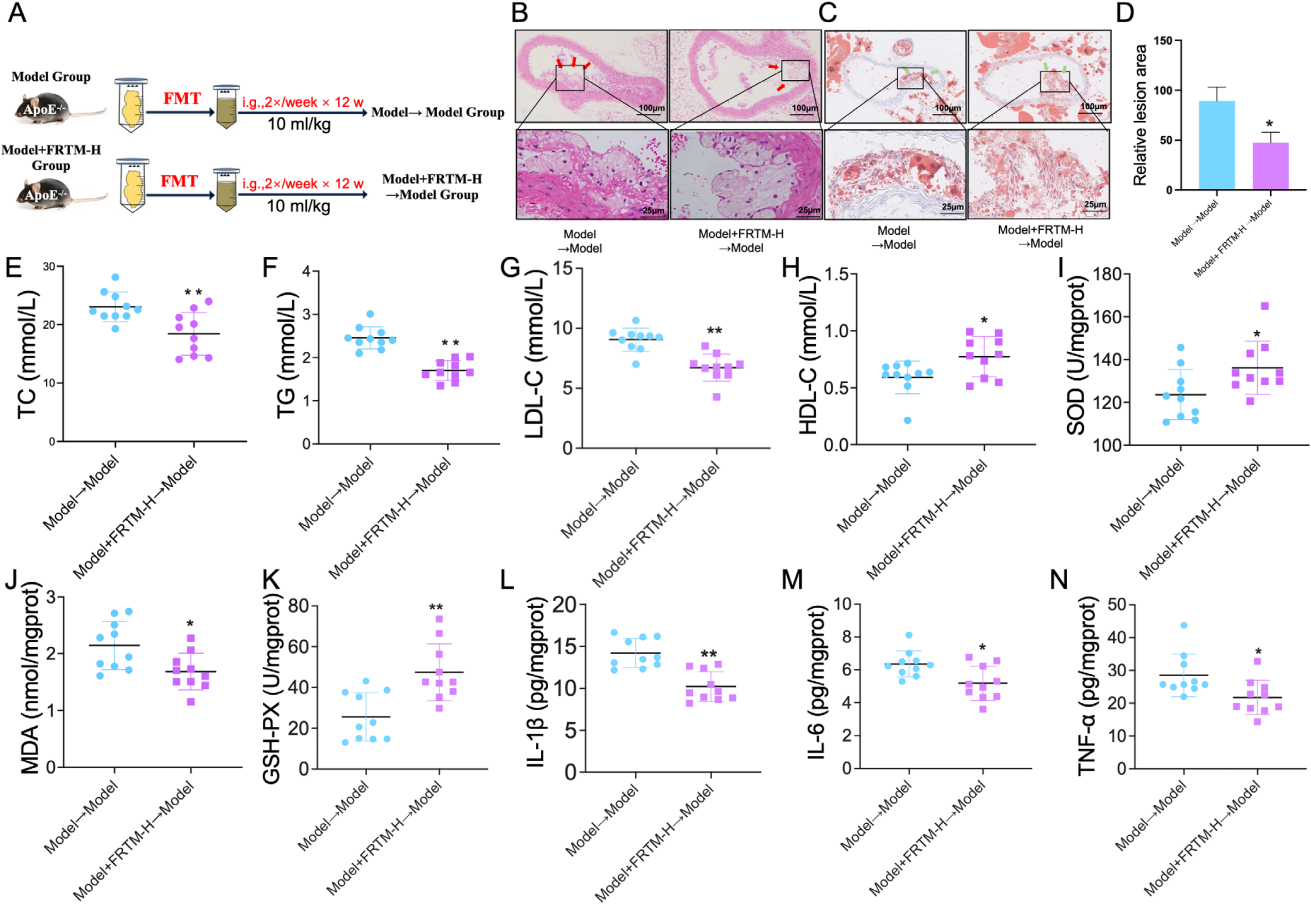
FMT from FRTM‐treated donors is associated with reduced aortic plaque burden and improved serum lipid profiles in AS mice. (A) Faecal Transplantation Grouping schematic. (B–D) HE staining showed that FRTM faecal transplantation could improve the pathological changes in the aorta (B) and oil‐red O staining indicated that FRTM faecal transplantation could decrease the plaque area and fat droplets in the aorta of AS mice (C, D). (E–H) FRTM faecal transplantation decreased the levels of TC (E) and TG (F) in serum of AS mice and improved the abnormal LDL‐C (G) and HDL‐C (H) levels in serum of AS mice. (I–K) FRTM faecal transplantation decreased the levels of SOD (I), MDA (J) and GSH‐PX (K) in serum of AS mice. (L–N) FRTM faecal transplantation significant reduction of IL‐1 β (L), IL‐6 (M) and TNF‐α (N) levels in serum of AS mice. Data are presented as the mean ± SD, *n* = 6 for pathological detection. *n* = 10 for biochemical testing. ^##^ *p* < 0.01 and ^#^ *p* < 0.05 as compared to the Control group; **p < 0.01 and *p < 0.05, as compared to the Model group. Representative histological images are shown, and quantitative analyses were performed by averaging multiple sections from six mice per group.

To explore whether microbiota alterations induced by FRTM might contribute to its protective effects, we performed FMT using faeces from FRTM‐H–treated donors (Figure 9A). Our histological examination and Oil Red O staining revealed varying degrees of improvement in the aortic lesion area in the Model+FRTM‐H → Model group compared to the Model→Model group (Figure 9B–D). Furthermore, the Model+FRTMH→Model group exhibited reduced serum levels of TC, TG and LDL‐C, while simultaneously increasing HDL‐C levels in AS mice, compared to the Model→Model group (Figure 9E–H). Additionally, the Model+FRTM‐H → Model group demonstrated enhanced activities of SOD and GSH‐Px, accompanied by decreased MDA levels in the serum of AS mice, as compared to the Model→Model group (Figure 9I–K). Notably, the Model+FRTM‐H → Model group also showed decreased levels of pro‐inflammatory factors in the serum of AS mice compared to the Model→Model group (Figure 9L–N).

### 3.9 FMT (Model+FRTM‐H → Model) is Associated With Improved Macrophage Polarization and Reduced Inflammation, Accompanied by Changes in PPARγ/NF‐κB and EP4/STAT3 Signalling

**FIGURE 10:**
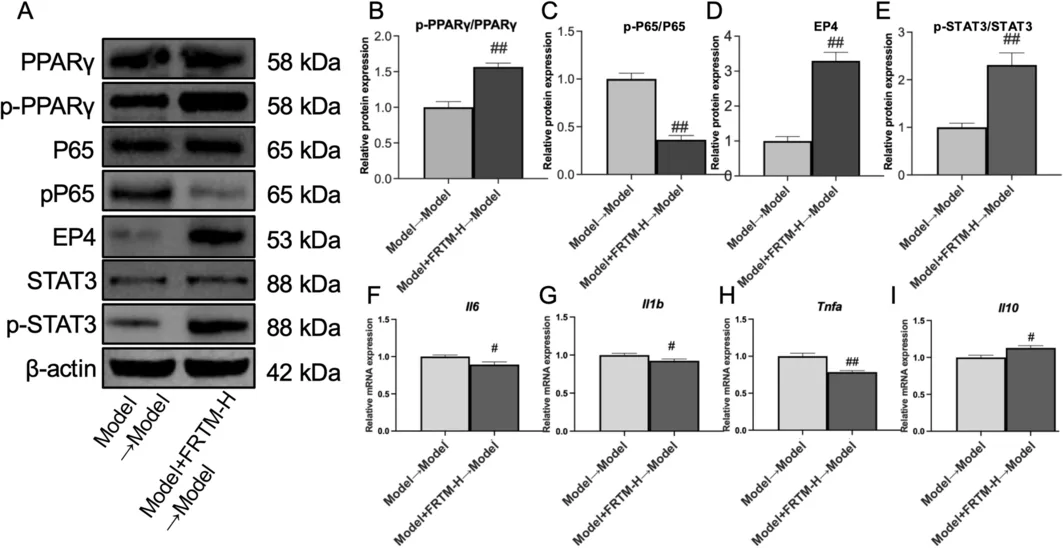
FMT from FRTM‐treated donors is associated with changes in PPARγ/NF‐κB and EP4/STAT3 signalling markers and inflammatory gene expression. (A–E) Western blot results showed that FRTM intervention increased the expression of p‐PPARγ/PPARγ and reduced the expression of p‐P65/P65; meanwhile, the expressions of EP4 and p‐STAT3/STAT3 were increased after FRTM intervention. (F–I) RT‐qPCR results showed that FRTM treatment reduced *Il1b, Il6* and *Tnfa* mRNA levels and increased *Il10* mRNA level. *n* = 3 per group.

**FIGURE 11:**
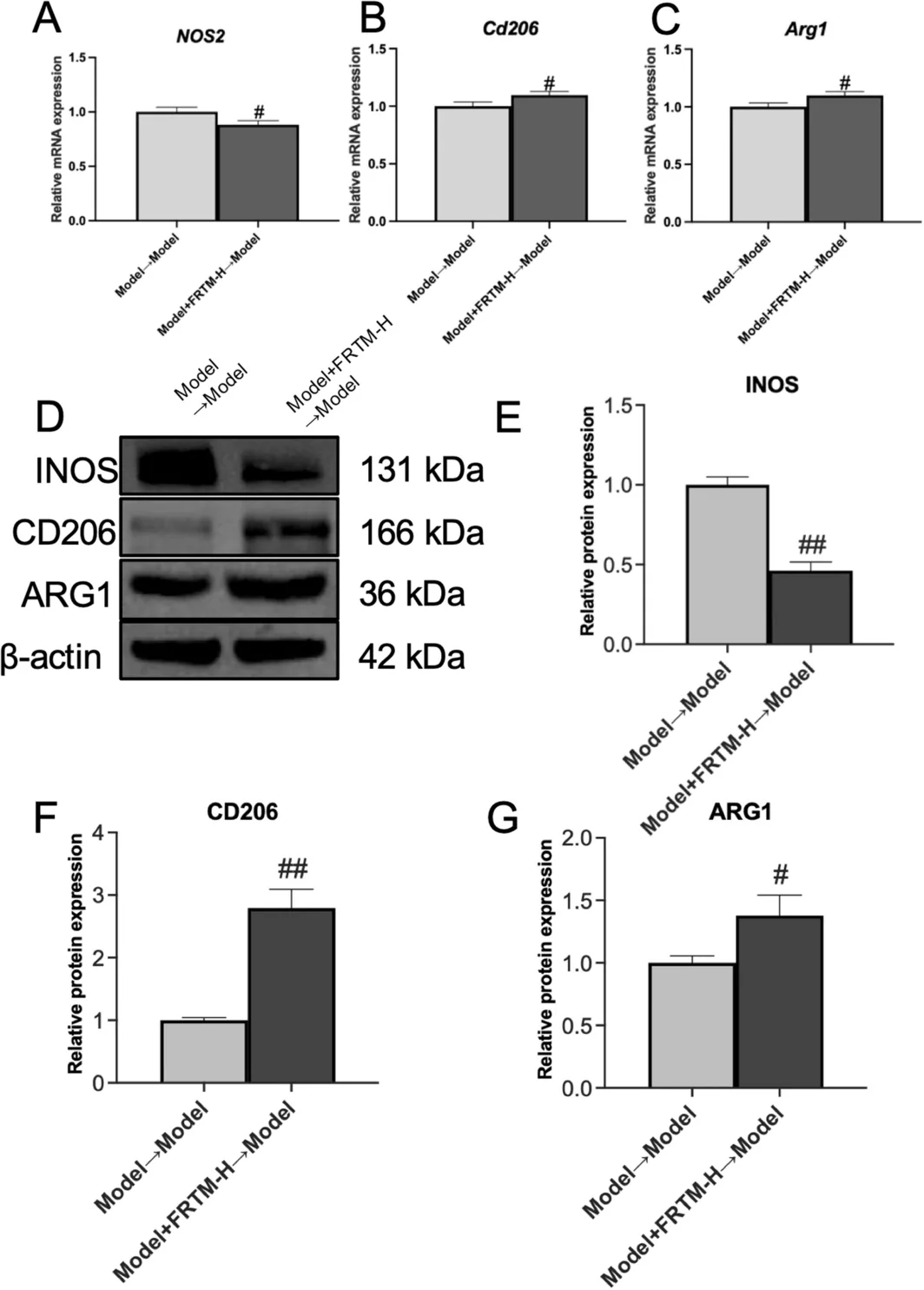
FRTM faecal transplantation regulated macrophage polarization. (A–C) RT‐qPCR results showed that FRTM faecal transplantation downregulated the expression of *Nos2* (A), while upregulating the expressions of *Cd206* (B) and *Arg1* (C). (D–G) Western blot showing that FRTM faecal transplantation downregulated the expression of INOS (E), while upregulating the expressions of CD206 (F) and ARG1 (G). *n* = 3 per group.

FMT from FRTM‐treated donors was associated with increased p‐PPARγ and decreased p‐P65 levels. In addition, compared with the Model→Model group, the Model+FRTM‐H → Model group showed higher EP4 expression and an increased p‐STAT3/STAT3 ratio (Figure 10A–E). RT‐qPCR further showed reduced Il1b, Il6 and Tnfa mRNA levels and increased Il10 expression (Figure 10F–I). Moreover, Nos2 expression was downregulated, whereas Cd206 and Arg1 expression were upregulated in recipient mice gavaged with faeces from FRTM‐treated donors compared with mice receiving faeces from the Model group (Figure 11A–C). Western blotting showed lower iNOS and higher CD206 and ARG1 levels in the FMT group (Figure 11D–G). These findings suggest that FMT from FRTM‐treated donors may partially reproduce the anti‐inflammatory phenotype observed after direct FRTM treatment.

## 4 Discussion

In our study, we established an AS mouse model by subjecting the animals to a high‐fat diet. After the model's completion, we observed lipid deposition in the arterial intima, leading to the formation of fatty streaks. This observation confirmed the successful establishment of the AS model [18]. Our results indicate that the administration of FRTM effectively reduced serum levels of TC, TG and LDL‐C in the mice. Simultaneously, it elevated levels of HDL‐C. Furthermore, FRTM treatment resulted in improvements in aortic pathology in the mice. For comparative purposes, we included atorvastatin, a commonly used medication in clinical practice for the prevention of AS, in our study [19]. Notably, we found that the therapeutic efficacy of high‐dose FRTM was comparable to that of atorvastatin, indicating substantial potential for FRTM as an AS treatment.

Emerging evidence underscores the pivotal role of gut microbiota in various pathological conditions, particularly cardiovascular diseases [20]. Our study demonstrates that the therapeutic effects of FRTM are closely linked to its ability to modulate gut microbiota. Specifically, FRTM intervention substantially decreases the relative abundance of *Turicibacter* while increasing the relative abundance of *Lactobacillus, Muribaculaceae, Bifidobacterium, Faecalibaculum* and *Alloprevotella* in our experimental setup.

These bacterial taxa are intricately linked to host metabolism and inflammation. *Turicibacter*, associated with dietary fat and weight changes, may influence energy homeostasis. Lactobacillus mitigates oxidative stress and inflammation and modulates cholesterol metabolism in ApoE^−/−^ mice [21]. Bifidobacterium reduces visceral fat and alleviates HFD‐induced obesity [22], with *Muribaculaceae* levels rising correspondingly [23]. *Faecalibaculum's* abundance correlates with short‐chain fatty acid (SCFA) production [24], which is critical for gut health and metabolic regulation [25]. *Alloprevotella* exhibits a negative correlation with lipid profiles and positively impacts glucose metabolism and insulin sensitivity through GLP‐1 secretion and beige fat induction [26]. Its fermentation byproducts, acetate and succinate, reinforce the gut barrier and suppress inflammation [27].

Metabolomics analysis provides a powerful tool to explore the metabolic characteristics of organisms during specific physiological or pathological states, offering insights into drug‐influenced metabolic pathways. In our study, serum untargeted metabolomics revealed that FRTM significantly impacts several metabolic pathways in AS mice, including AA metabolism, pyrimidine metabolism and steroid hormone biosynthesis. Research indicates that over 30% of human metabolites may originate from gut microbiota, which can influence host health and disease [28, 29]. Our Spearman's correlation analysis identified significant correlations between specific gut bacteria (e.g., *Turicibacter*, *Lactobacillus*, *Muribaculaceae*, *Bifidobacterium*, *Faecalibaculum* and *Alloprevotella*) and metabolites. By integrating metabolomics data with 16S rRNA sequencing results, we found that AA metabolism is a shared pathway influenced by both gut microbiota and FRTM. This suggests that FRTM may modulate gut microbiota to regulate AA metabolism, thereby exerting therapeutic effects on AS.

Arachidonic acid (AA), an omega‐6 polyunsaturated fatty acid, is metabolized into bioactive prostaglandins (PGs) and thromboxanes (TXs), which play critical roles in the progression of AS [30]. In AS, macrophages polarize into pro‐inflammatory M1 macrophages, which exacerbate inflammation and promote plaque instability, or anti‐inflammatory M2 macrophages, which facilitate inflammation resolution and tissue repair [31]. AA metabolites, such as PGE2 and PGJ2, modulate macrophage polarization toward the M2 phenotype through activation of the PPARγ and EP4/STAT3 signalling pathways, thereby reducing inflammation and atherosclerotic plaque formation [32]. Specifically, PGJ2 acts as a PPARγ ligand to inhibit NF‐κB activation, reducing the production of pro‐inflammatory cytokines such as TNF‐α and IL‐6, while PGE2 promotes STAT3 phosphorylation via the EP4 receptor, enhancing the expression of anti‐inflammatory cytokines like IL‐10 [33]. Our findings demonstrate that FRTM increases anti‐inflammatory PGs (e.g., PGE2, PGJ2) and decreases pro‐inflammatory metabolites (e.g., PGG2, TXB2), thereby mitigating AS pathology through modulation of AA metabolism and associated signalling pathways, highlighting its therapeutic potential [34]. Several mechanisms may explain how FRTM influences AA metabolism. First, FRTM constituents may directly modulate AA‐metabolizing enzymes: ferulic acid from Angelica sinensis and puerarin from *Pueraria lobata* have been reported to affect eicosanoid biosynthesis [35]. Second, FRTM‐induced gut microbiota remodelling may indirectly regulate host AA metabolism through microbial metabolites such as short‐chain fatty acids and secondary bile acids, which influence inflammatory signalling and lipid mediator profiles [36]. Third, taxa enriched by FRTM (e.g., Lactobacillus and Bifidobacterium) have been shown to modulate host fatty acid metabolism via microbe–host crosstalk [37]. We did not directly measure AA or its precursors in FRTM preparations, nor did we dissect the relative contributions of the direct pharmacological versus microbiota‐mediated effects. Future studies using germ‐free models, antibiotic pretreatment, or in vitro fermentation combined with targeted lipidomics will help clarify these possibilities.

Our data suggest that FRTM is associated with changes in signalling pathways implicated in inflammation and macrophage polarization, including PPARγ/NF‐κB and EP4/STAT3 [38]. The observed increase in p‐PPARγ and decrease in p‐P65 are consistent with reduced inflammatory signalling, but they do not prove that these pathways are required for the protective effect of FRTM. Similarly, the changes in Nos2, Cd206, Arg1, iNOS, CD206 and ARG1 suggest a shift toward an M2‐like macrophage phenotype in FRTM‐treated mice; however, direct functional validation is still needed to establish whether this shift is driven by PPARγ/NF‐κB or EP4/STAT3 signalling. PPARγ, a nuclear receptor expressed in various tissues, plays a crucial role in transcriptional regulation, influencing cellular functions and metabolism [38]. Activating PPARγ can suppress NF‐κB activity, reducing the production of inflammatory cytokines such as TNF‐α, IL‐1β and IL‐6 28. NF‐κB, a key transcription factor, typically resides in the cytoplasm but translocates to the nucleus upon activation, triggering inflammation‐related gene transcription [39]. FRTM's ability to activate PPARγ and inhibit NF‐κB signalling underscores its anti‐inflammatory potential [40]. FRTM also impacts the EP4/STAT3 pathway. PGE2 activation of the EP4 receptor promotes STAT3 phosphorylation, enhancing anti‐inflammatory cytokine expression (e.g., IL‐10) and reducing pro‐inflammatory cytokines [41]. Our findings show that FRTM increases p‐STAT3 levels, indicating its role in modulating this pathway to suppress inflammation [42]. Macrophage polarization is a key factor in AS progression. M1 macrophages promote inflammation, while M2 macrophages resolve it [43]. FRTM shifts macrophage polarization from M1 to M2 by activating PPARγ and EP4/STAT3, reducing inflammation and plaque formation [44]. Dysregulation of these pathways can lead to excessive M1 polarization and inflammation, exacerbating AS. FRTM's modulation of these pathways offers a promising strategy for treating AS by restoring macrophage polarization balance and reducing inflammation [43].

In our study, FMT was employed to investigate the role of gut microbiota during FRTM treatment for AS. Our results demonstrated that faecal samples from FRTM‐treated mice could correct the imbalance in macrophage polarization associated with AS, thereby reducing inflammation through modulation of the PPARγ/NF‐κB and EP4/STAT3 pathways. This highlights FMT as a powerful tool for exploring the interactions between herbal medicines, gut microbiota and host metabolism. Previous studies have shown that FMT can effectively rebalance gut microbiota, offering a potential therapeutic strategy for various conditions [45]. Moreover, introducing pro‐inflammatory microbiota into mice has been shown to enhance systemic inflammation and accelerate atherogenesis [46]. In our FMT experiments, transplanting faecal matter from FRTM‐treated mice increased the abundance of beneficial bacteria such as *f_Coriobacteriaceae, f_S24‐7, f_Lachnospiraceae* and *f_Adlercreutzia*, while concurrently reducing inflammation associated with AS [47]. However, because the recipients were not antibiotic‐depleted or germ‐free, donor engraftment was likely incomplete. Therefore, the FMT findings should be interpreted as supportive rather than definitive evidence of causality.

Dysregulation of the PPARγ/NF‐κB and EP4/STAT3 pathways in AS is associated with excessive M1 macrophage activation and impaired M2 macrophage function, thereby contributing to plaque development and disease progression. In the present study, FRTM treatment was accompanied by improved macrophage polarization and reduced inflammatory status, together with changes in PPARγ/NF‐κB and EP4/STAT3 pathway markers, suggesting that these pathways may be involved in the observed protective effects. Notably, faecal microbiota transplantation (FMT) from FRTM‐treated donors partially recapitulated these benefits, supporting a potential contributory role of gut microbiota. However, because we did not perform functional validation (e.g., pharmacological inhibition, siRNA‐mediated knockdown or genetic loss‐of‐function approaches) and recipients were not preconditioned with antibiotic depletion or germ‐free conditions prior to FMT, these findings should be interpreted as associative rather than definitive evidence of mechanistic requirement. One limitation is that aortic sections were taken at plaque‐bearing levels rather than the three‐leaflet aortic valve sinus plane, the recognized standard for plaque quantification in ApoE^−/−^ mice; future studies will adopt the valve sinus protocol. Overall, our data indicate that FRTM is a promising candidate for AS intervention, and future studies incorporating pathway‐specific perturbations and microbiota‐conditioned models are warranted to establish causality and assess long‐term efficacy and translational potential.

## 5 Conclusion

**FIGURE 12:**
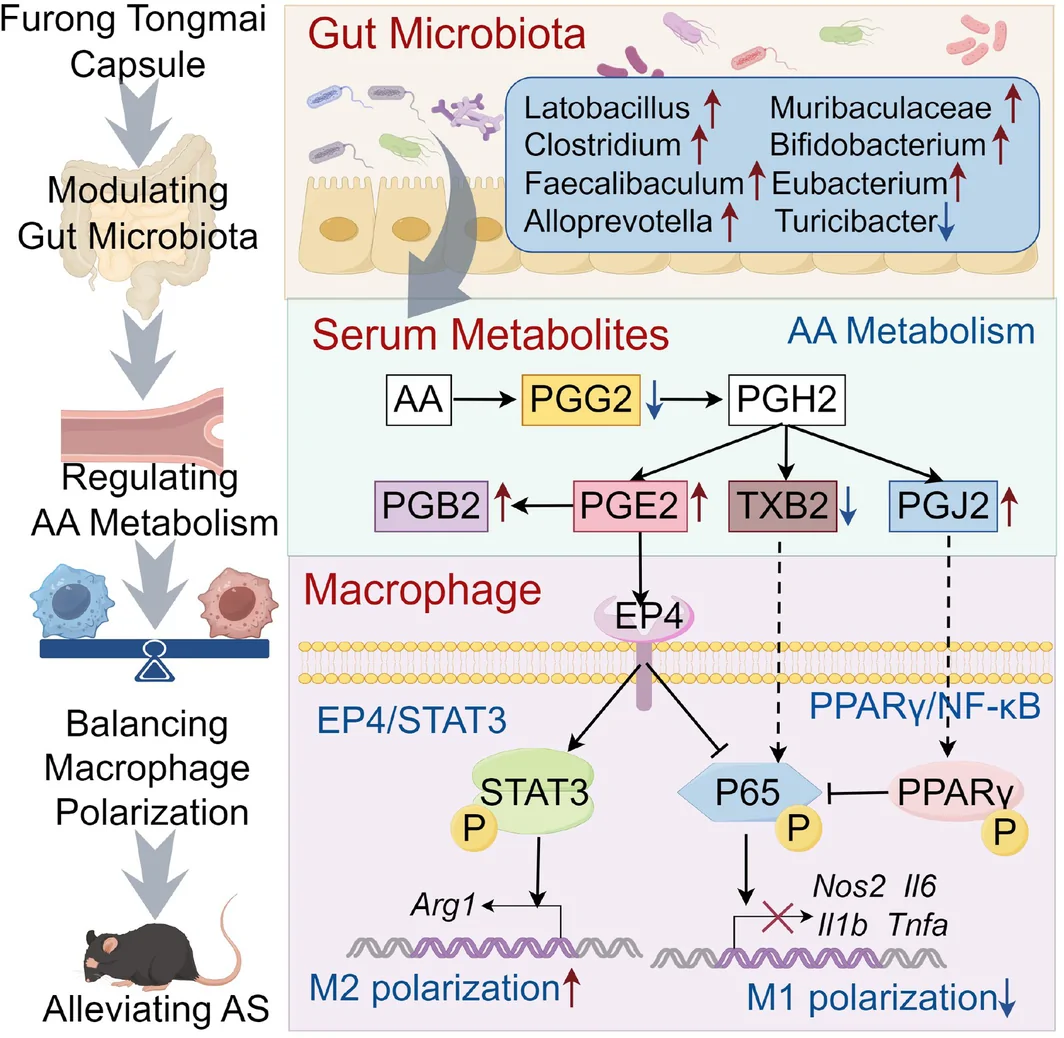
FRTM can rectify the imbalance in macrophage polarization in AS mice through the PPARγ/NF‐κB pathway and the EP4/STAT3 pathway, thereby alleviating inflammatory responses and exerting anti‐atherosclerotic effects.

In this preclinical study, FRTM improved serum lipid profiles and aortic pathology in ApoE^−/−^ mice. Integrated microbiota and metabolomic analyses suggest that these effects may be linked to arachidonic acid metabolism. FRTM treatment was associated with altered macrophage polarization and changes in PPARγ/NF‐κB and EP4/STAT3 signalling, together with reduced inflammatory responses (Figure 12). However, because these findings are primarily associative, additional functional studies are needed to determine whether these pathways are causally involved in the anti‐atherosclerotic effects of FRTM.

## Author Contributions

**Feng Wang:** investigation, visualization. **Hanzhou Li:** writing – original draft, investigation, validation, data curation. **Huantian Cui:** writing – review and editing, conceptualization. **Jing Yang:** investigation, software. **Lirong Fan:** validation, visualization. **Xiuxiu Sang:** formal analysis, visualization, validation. **Ziang Ma:** visualization, methodology. **Shuquan Lv:** writing – original draft, investigation. **Yuhong Bian:** conceptualization, supervision. **Yuming Wang:** investigation, formal analysis, writing – original draft. **Lixin Wang:** validation.

## Funding

The authors have nothing to report.

## Conflicts of Interest

The authors declare no conflicts of interest.

## Supporting information


**Table S1:** Elution gradient table.
**Figure S1:** The chemical profiles of FRTM using UPLC–MS.
**Table S2:** Primer sequence.
**Table S3:** Differential metabolites.

## Data Availability

The data that support the findings of this study are available from the corresponding author upon reasonable request.
