# Supplementary material for: Furong Tongmai Capsule Ameliorates Atherosclerosis in ApoE −/− Mice by Modulating Gut Microbiota, Arachidonic Acid Metabolism and Macrophage Polarization
Source: J Cell Mol Med. 2026 Sep 7;30(17):e71354. doi: 10.1111/jcmm.71354 (PMC13550762; doi:10.1111/jcmm.71354)
Supplement: Supplementary file 1 [file JCMM-30-e71354-s001.docx]

**1 Reagents**

Fu-Rong-Tong-Mai capsule (Lot:Z20070041) was purchased from Cangzhou Hospital of Intergrted TCM-WM Hebei. Atorvastatin Calcium Tablets (Lot: LW4386) was purchased from Pfizer Inc. Triglycerides (cat: A110-1-1), total cholesterol (cat: A111-1-1), high-density lipoprotein cholesterol (cat: A112-1-1), low-density lipoprotein cholesterol (cat: A113-1-1), superoxide dismutase (cat: A001-3-2), glutathione peroxidase (cat: A005-1-2), malondialdehyde (cat: A003-4-1) kits were all purchased from Nanjing Jiancheng Biological Engineering Institute. Elisa kits for IL-1β (cat: ml301814), IL-6 (cat: ml098430), TNF-α (cat: ml002095) were purchased from Shanghai Enzyme Research Biotechnology Co., Ltd. Primary antibodies for PPAR Gamma (16643-1-AP), NF-κB p65 (10745-1-AP), phospho-NF-κB p65 (82335-1-RR), STAT3 (10253-2-AP), iNOS (22226-1-AP), CD206 (18704-1-AP), Arginase-1 (16001-1-AP) and Beta Actin (20536-1-AP) were purchased from Proteintech Group, Inc. Primary antibodies for phospho-PPAR Gamma (bs-3737R), EP4 (bs-8538R) phospho-STAT3 (bs-3429R) were purchased from Beijing Bioss Co., Ltd.

**2 UPLC-MS analysis of** **QGMS**

FRTM was obtained from Cangzhou Hospital of Integrated Traditional Chinese Medicine and Western Medicine. Ultra-performance liquid chromatography (UPLC) coupled with mass spectrometer (MS) was conducted as the quality control of FRTM.

**2.1 Sample preparation and extraction**

2.1.1 Take a certain mass of powder, add methanol, ultrasonic dissolution for 1 h.

2.1.2 Take 100 μL solution and dilute it with 50 % methanol to 100 μg / mL.

2.1.3 The centrifuge tube was placed in a low-temperature centrifuge and centrifuged at 4 °C, 12000 rpm for 5 min.

2.1.4 A total of 100 μL supernatant was placed in the injection bottle for mass spectrometry detection.

**2.2 HPLC Conditions**

Column: Waters UPLC HSS T3 (1.8 μm, 2.1 mm × 100 mm)

Mobile Phase: A phase (water + 0.1% formic acid), B phase (methanol)

Gradient Elution: Refer to Table S1

Flow Rate: 0.3 mL/min

Injection Volume: 10.0 µL

Column Temperature: 40°C

**Table S1** Elution gradient table

| Time（min） | Mobile Phase | |
| --- | --- | --- |
|  | A（v%） | B（v%） |
| 0 | 98 | 2 |
| 1.0 | 98 | 2 |
| 10.0 | 0 | 100 |
| 14.0 | 0 | 100 |
| 14.1 | 98 | 2 |
| 16.0 | 98 | 2 |

**2.3 MS Conditions**

Mass spectrometric analysis was performed using a quadrupole orbitrap mass spectrometer equipped with a heated electrospray ionization source (Q Exactive™). The ion source voltages for both positive and negative ions were set at 3.7 kV and 3.5 kV, respectively. The capillary temperature was maintained at 320°C. Sheath gas pressure was set at 30 psi, and auxiliary gas pressure was set at 10 psi. The solvent heated-evaporative gas temperature was 300°C. Both sheath and auxiliary gases were nitrogen, while the collision gas was also nitrogen with a pressure of 1.5 mTorr. For the primary full scan, the parameters were as follows: resolution 70000, automatic gain control target 1×10^6^, maximum injection time 50 ms, mass-to-charge ratio scan range 100–1500. Mass axis calibration was achieved using an external standard method with a mass error of 5 ppm. Positive ion calibration standards were 784.4691, 194.05791, 364.13695, 494.17881, 416.17073, 868.48204, 418.12638, and 784.46091. Metabolite identification utilized a dd-MS2 scan mode (data-dependent scan mode) with the following parameters: resolution 17500, automatic gain control target 1×10^5^, maximum injection time 50 ms, up to 10 ions in the second-level fragmentation (dynamically excluded), mass isolation window 2, collision energy 30 V, intensity threshold 1×10^5^. The Thermo Xcalibur 2.2 SP1.48 software controlled the liquid chromatography-mass spectrometry system and facilitated data acquisition.

**2.4 UPLC-MS analysis results of** **FRTM decoction**

We found that Astragaloside A, Ferulic Acid, Harpagide, Harpagoside, Puerarin, Dioscin, Liquiritin and Glycyrrhizic acid in FRTM (**Figure. S1**).

**Figure. S1.** The chemical profiles of FRTM using UPLC-MS.

**3 16S rRNA Sequencing**

**3.1 Fecal Genomic DNA Extraction**

Total genomic DNA from the cecal contents of mice was extracted using the CTAB/SDS method. The DNA concentration and purity were assessed using a 1% agarose gel. Based on the concentration, DNA was diluted to 1 ng/µL with sterile water.

**3.2 16S rRNA Gene PCR Amplification and Sequencing**

The V3-V4 region of the 16S rRNA gene was amplified using the primers 338F (5'-ACTCCTACGGGAGGCAGCAG-3') and 806R (5'-GGACTACHVGGGTWTCTAAT-3'). The PCR amplification system included: 10 ng of template DNA, 0.2 µM of forward and reverse primers, and 15 µL of Phusion® High-Fidelity PCR Master Mix (New England Biolabs). The reaction conditions were: initial denaturation at 98°C for 1 minute; followed by 15 cycles of denaturation at 95°C for 10 seconds, annealing at 50°C for 30 seconds, and extension at 72°C for 30 seconds; with a final extension at 72°C for 5 minutes and storage at 4°C. The mixed PCR products were purified using the Qiagen Gel Extraction Kit (Qiagen, Germany). Detection was performed using 2% agarose gel electrophoresis. Sequencing libraries were generated using the TruSeq® DNA PCR-Free Sample Preparation Kit (Illumina, USA), and the library quality was assessed using the Qubit@ 2.0 Fluorometer (Thermo Scientific) and the Agilent Bioanalyzer 2100 system. Finally, sequencing of the libraries was performed on the Illumina NovaSeq platform, yielding 250 bp paired-end sequences.

**3.3 Sequencing Data Analysis**

Diversity analysis was conducted on the sequencing data results. Alpha diversity of the gut microbiota in each group was assessed using the Shannon and Simpson indices. Beta diversity analysis results were represented through Principal Coordinate Analysis (PCoA), which is an unconstrained dimensionality reduction method that can display the similarity and differences in community composition among different sample groups. Inter-group differences in diversity indices were tested using the Wilcoxon Rank-Sum Test. Additionally, OTUs were classified at the species level by comparison with databases, and species abundance analysis was performed for each group's samples at the phylum and genus levels. Finally, the Phylogenetic Investigation of Communities by Reconstruction of Unobserved States (PICRUSt) database was used to analyze and predict the related metabolic pathways that the differentially abundant microbial communities might influence.

**4. Metabolomics Analysis**

**4.1 Serum Sample Processing**

Take 100 μL of serum sample and add 400 μL of 80% methanol aqueous solution. Vortex to mix, then place on ice for 5 minutes, followed by centrifugation at 20,000 g for 20 minutes at 4°C. After centrifugation, dilute the supernatant with ultra-pure water to a methanol concentration of 53%. Centrifuge again at 20,000 g for 20 minutes at 4°C, collect the supernatant as the sample for testing. Mix equal amounts of all samples to create a QC (Quality Control) sample, which is periodically analyzed throughout the process to monitor instrument stability.

**4.2 Chromatography and Mass Spectrometry Conditions**

Select a Hypesil Gold column (C18) chromatographic column (2.1 mm × 100 mm, 1.9 μm). The mobile phase consists of (A) 0.1% formic acid and (B) methanol, with gradient elution as follows: 0 min, 98% A, 2% B; 1.5 min, 98% A, 2% B; 12 min, 0% A, 100% B; 14 min, 0% A, 100% B; 14.1 min, 98% A, 2% B; 17 min, 98% A, 2% B. The column temperature is set to 40°C, the flow rate is 0.2 mL/min, and the injection volume is 2 μL. Mass spectrometry conditions: Use an electrospray ion source (ESI) to detect both positive and negative ion modes simultaneously. The ESI source settings are as follows: Spray Voltage: 3.2 kV; Sheath gas flow rate: 40 arb; Aux Gas flow rate: 10 arb; Capillary Temperature: 320°C. Polarity: positive; negative; the mass-to-charge (m/z) scanning range is selected from 100 to 1500; MS/MS secondary scanning is data-dependent.

**4.3 Data Processing and Analysis**

Employ high-resolution mass spectrometry (HRMS) detection technology and utilize databases such as mzCloud (https://www.mzcloud.org/), mzVault, and MassList, along with Compound Discoverer 3.1 (CD3.1, Thermo Fisher) software for peak identification, retention time correction, and automatic integration as part of the preprocessing. A visual matrix is obtained that includes metabolite numbers, retention times, m/z, metabolite names, ion modes, and peak areas. The peak area of each characteristic peak represents the relative quantitative value of a metabolite. The quantitative results are normalized using the total peak area, yielding the final quantitative results of the metabolites. Subsequently, multivariate statistical analysis of the metabolites is performed, including principal component analysis (PCA) and partial least squares-discriminant analysis (PLS-DA), to reveal differences in metabolic patterns between different groups. Metabolite correlation analysis is used to reveal relationships between samples and among metabolites. Finally, functional analysis such as metabolic pathway analysis is conducted to explain the biological significance of the metabolites. This experiment screens potential biomarkers based on P ≤ 0.05 and VIP (Variable importance of projection) where VIP > 1. Differential metabolites with a Fold change (FC) > 1.2 or FC < 0.80 are subjected to metabolic pathway enrichment analysis based on KEGG data (https://www.kegg.jp/).

**Table S2 Primer sequence**

| **Genes** | **Primer sequence (5’-3’)** |
| --- | --- |
| ***Actb*** | Forward: GATATCGCTGCGCTGGTCG |
|  | Reverse: CATTCCCACCATCACACCCT |
| ***Il1b*** | Forward: ATGCCACCTTTTGACAGTGATG |
|  | Reverse: TGTGCTGCTGCGAGATTTGA |
| ***Il6*** | Forward: AGCCAGAGTCCTTCAGAGAGA |
|  | Reverse: GCCACTCCTTCTGTGACTCC |
| ***Tnfa*** | Forward: GATCGGTCCCCAAAGGGATG |
|  | Reverse: CCACTTGGTGGTTTGTGAGTG |
| ***Il10*** | Forward: ACCTGGTAGAAGTGATGCCC |
|  | Reverse: ACACCTTGGTCTTGGAGCTTAT |
| ***Nos2*** | Forward: GAGCAACTACTGCTGGTGGT |
|  | Reverse: CGATGTCATGAGCAAAGGCG |
| ***Cd206*** | Forward: ATGGATTGCCCTGAACAGCA |
|  | Reverse: TGTACCGCACCCTCCATCTA |
| ***Arg1*** | Forward: ACATTGGCTTGCGAGACGTA |
|  | Reverse: ATCACCTTGCCAATCCCCAG |

**Table S3 Differential metabolites**

| **No.** | **Formula** | **RT [min]** | **m/z** | **Metabolites** | **VIP** | | **FC** | | **Trend** | | | **Pathway** | |
| --- | --- | --- | --- | --- | --- | --- | --- | --- | --- | --- | --- | --- | --- |
|  |  |  |  |  | **M vs. C** | **H vs. M** | **M vs. C** | **H vs. M** | **M vs. C** | **H vs. M** |  | |  |
| 1 | C21 H32 O3 | 7.813 | 331.22769 | 17α-Hydroxypregnenolone | 2.87893727 | 1.136300298 | 9.465968466 | 0.236431563 | ↑## | ↓# |  | |  |
| 2 | C32 H58 N O7 P | 10.221 | 600.40247 | PC (22:4e/2:0) | 2.197686591 | 1.073678467 | 0.300990848 | 2.475417596 | ↓## | ↑## |  | |  |
| 3 | C32 H58 N O7 P | 10.225 | 644.39417 | LPC 24:4 | 2.212337923 | 1.039256953 | 0.298692046 | 2.31302429 | ↓## | ↑# |  | |  |
| 4 | C15 H22 N6 O7 | 7.88 | 399.16107 | ENH | 2.183487109 | 1.344305658 | 3.345594493 | 0.240478884 | ↑## | ↓# |  | |  |
| 5 | C15 H24 O4 | 6.705 | 267.16016 | 3-[4-methyl-1-(2-methylpropanoyl)-3-oxocyclohexyl]butanoic acid | 1.444881671 | 1.120939745 | 4.222542089 | 0.13273573 | ↑## | ↓## |  | |  |
| 6 | C27 H44 O2 | 10.329 | 401.34055 | 7-Ketocholesterol | 1.66157403 | 1.012010717 | 0.322085704 | 2.596293299 | ↓## | ↑# |  | |  |
| 7 | C10 H18 O2 | 6.015 | 169.12367 | cis-2-Decenoic acid | 1.139890164 | 1.377596218 | 3.171060752 | 0.05548498 | ↑## | ↓## |  | |  |
| 8 | C24 H40 O4 | 7.807 | 437.29099 | Chenodeoxycholic Acid | 1.707556629 | 1.048797666 | 0.168976932 | 4.427443351 | ↓## | ↑# |  | |  |
| 9 | C12 H23 N O4 | 6.344 | 244.15527 | 2-Methylbutyroylcarnitine | 1.410439248 | 1.163390184 | 2.969835137 | 0.283052995 | ↑# | ↓## |  | |  |
| 10 | C13 H24 O5 | 6.156 | 243.15889 | 3-hydroxy-2-octylpentanedioic acid | 1.497003177 | 1.224959927 | 2.815994022 | 0.221570517 | ↑# | ↓# |  | |  |
| 11 | C10 H7 N O4 | 5.341 | 206.04475 | Xanthurenic Acid | 1.212338562 | 1.310288138 | 3.133902939 | 0.110444611 | ↑# | ↓# |  | |  |
| 12 | C20 H32 O5 | 6.275 | 375.21387 | Prostaglandin E2 | 2.291274967 | 1.403438033 | 0.484233964 | 6.022663257 | ↓## | ↑## | B | |  |
| 13 | C20 H30 O4 | 7.498 | 315.19647 | Prostaglandin J2 | 2.030170068 | 1.686784461 | 0.22440814 | 7.009177487 | ↓## | ↑## | B | |  |
| 14 | C19 H34 O4 | 7.825 | 365.20798 | 1,2-dihydroxyheptadec-16-yn-4-yl acetate | 2.291274967 | 1.403438033 | 1.999215679 | 0.634316588 | ↑## | ↓# |  | |  |
| 15 | C7 H7 N O2 | 1.391 | 138.05519 | Trigonelline | 1.756400718 | 1.484036297 | 1.95227671 | 0.407497507 | ↑## | ↓## |  | |  |
| 16 | C24 H40 O4 | 7.853 | 391.28543 | Deoxycholic Acid | 1.770189264 | 1.0600591 | 0.208964853 | 3.566033393 | ↓## | ↑# |  | |  |
| 17 | C12 H20 O8 | 8.267 | 293.11932 | (3R)-4,4-Dimethyl-2-oxotetrahydro-3-furanyl beta-D-glucopyranoside | 1.539092636 | 1.244201993 | 7.674528269 | 0.087402255 | ↑## | ↓# |  | |  |
| 18 | C18 H30 O3 | 7.416 | 295.22531 | 9-HOTrE | 1.499767529 | 1.439914449 | 1.886222679 | 0.559004176 | ↑## | ↓# |  | |  |
| 19 | C20 H32 O6 | 7.662 | 367.21381 | Prostaglandin G2 | 1.212338562 | 1.484036297 | 2.74945585 | 0.318122332 | ↑# | ↓## | B | |  |
| 20 | C6 H13 O9 P | 1.583 | 259.02219 | D-Glucose 6-phosphate | 2.183487109 | 1.537206359 | 2.451316611 | 0.354105223 | ↑## | ↓## |  | |  |
| 21 | C16 H30 O2 | 9.466 | 253.21698 | Palmitoleic Acid | 1.807451796 | 1.308368837 | 8.176620053 | 0.126978786 | ↑ | ↓ |  | |  |
| 22 | C14 H28 O2 | 6.619 | 227.20174 | Myristic Acid | 2.197686591 | 1.17422117 | 3.866871419 | 0.139704911 | ↑ | ↓ |  | |  |
| 23 | C19 H28 O2 | 8.869 | 287.20114 | Dehydroepiandrosterone | 1.539092636 | 1.346257936 | 0.152293063 | 4.739360954 | ↓## | ↑## | E | |  |
| 24 | C10 H12 N4 O6 | 4.695 | 283.06863 | Xanthosine | 1.444881671 | 1.440610583 | 0.485402391 | 2.049911467 | ↓# | ↑# |  | |  |
| 25 | C2 H8 N O4 P | 1.329 | 102.05627 | O-Phosphorylethanolamine | 2.212337923 | 1.488673061 | 1.642597715 | 0.471276409 | ↑# | ↓## |  | |  |
| 26 | C21 H32 O2 | 8.116 | 317.24692 | Pregnenolone | 1.096536966 | 1.61947751 | 0.475820082 | 2.007847333 | ↓## | ↑## | E | |  |
| 27 | C20 H30 O4 | 7.087 | 333.20703 | Prostaglandin B2 | 2.87893727 | 1.530069149 | 0.148977956 | 7.672971082 | ↓## | ↑## | B | |  |
| 28 | C20 H34 O6 | 6.261 | 369.22821 | Thromboxane B2 | 1.410439248 | 1.244637404 | 2.725882556 | 0.174739748 | ↑# | ↓## | B | |  |
| 29 | C22 H34 O4 | 7.318 | 361.23798 | (±)19(20)-DiHDPA | 1.807451796 | 1.658388483 | 1.607751907 | 0.634869966 | ↑## | ↓# |  | |  |
| 30 | C18 H32 Ca N2 O10 | 9.574 | 475.16345 | Calcium D-Panthotenate | 2.042546504 | 1.820789404 | 1.841197279 | 0.497490142 | ↑## | ↓## |  | |  |
| 31 | C10 H18 O4 | 5.931 | 203.12782 | 2-[5-(2-hydroxypropyl)oxolan-2-yl]propanoic acid | 1.096536966 | 1.41106755 | 2.745772329 | 0.065737653 | ↑## | ↓# |  | |  |
| 32 | C22 H40 N O4 | 6.236 | 382.29489 | ACar 15:2 | 1.420979908 | 1.244637404 | 3.622037085 | 0.14233217 | ↑## | ↓# |  | |  |
| 33 | C26 H36 O4 | 7.026 | 413.2663 | 5-[(10Z)-14-(3,5-dihydroxyphenyl)tetradec-10-en-1-yl]benzene-1,3-diol | 1.720457177 | 1.247699814 | 0.319984489 | 2.045906764 | ↓## | ↑# |  | |  |
| 34 | C11 H12 O3 | 8.584 | 188.12811 | 4-oxo-5-phenylpentanoic acid | 2.030170068 | 1.111325392 | 3.628789343 | 0.306848772 | ↑## | ↓## |  | |  |
| 35 | C10 H14 N2 O5 | 4.869 | 241.08308 | Thymidine | 1.497003177 | 1.475570396 | 1.54827393 | 0.327591531 | ↑# | ↓## | C | |  |
| 36 | C9 H12 N2 O5 | 2.775 | 227.06755 | 2-Deoxyuridine | 1.139890164 | 1.384530613 | 1.52368983 | 0.731002749 | ↑## | ↓# |  | |  |
| 37 | C5 H6 N2 O2 | 4.847 | 127.05049 | Thymine | 1.66157403 | 1.802780355 | 1.640939293 | 0.251576403 | ↑# | ↓## | C | |  |
| 38 | C26 H45 N O7 S | 7.913 | 514.28418 | Taurocholic acid | 1.707556629 | 1.120939745 | 3.855274729 | 0.198805558 | ↑# | ↓# |  | |  |
| 39 | C24 H40 O4 | 7.807 | 437.29099 | Chenodeoxycholic Acid | 2.042546504 | 1.663601456 | 5.917967534 | 0.192607668 | ↑# | ↓# |  | |  |
| 40 | C24 H40 O4 | 7.853 | 391.28543 | Deoxycholic Acid | 1.770189264 | 1.654428785 | 4.785493766 | 0.171468738 | ↑# | ↓# | C | |  |
| 41 | C24 H40 O3 | 9.126 | 375.29077 | Lithocholic Acid | 1.499767529 | 1.423144483 | 2.272164322 | 0.470335441 | ↑## | ↓## |  | |  |

Note: Control, Model and FRTM-H groups. #: p < 0.05, compared with the control group. ##: p < 0.01, compared with the control group. *

: p < 0.05, compared with the Model group. **: p < 0.01, compared with the Model group.

Abbreviations: B：Arachidonic acid metabolism；C：Pyrimidine metabolism; E：Steroid hormone biosynthesis;
